# Reaction mechanism and kinetics for CO_2_ reduction on nickel single atom catalysts from quantum mechanics

**DOI:** 10.1038/s41467-020-16119-6

**Published:** 2020-05-07

**Authors:** Md Delowar Hossain, Yufeng Huang, Ted H. Yu, William A. Goddard III, Zhengtang Luo

**Affiliations:** 1 Department of Chemical and Biological Engineering, William Mong Institute of Nano Science and Technology, and Hong Kong Branch of Chinese National Engineering Research Center for Tissue Restoration and Reconstruction, The Hong Kong University of Science and Technology, Clear Water Bay, Kowloon, Hong Kong; 2 Materials and Process Simulation Center (mc 134-74), California Institute of Technology, Pasadena, CA 91125 USA; 3 Department of Chemical Engineering, California State University, Long Beach, CA 90840 USA

**Keywords:** Electrocatalysis, Electrocatalysis, Atomistic models

## Abstract

Experiments have shown that graphene-supported Ni-single atom catalysts (Ni-SACs) provide a promising strategy for the electrochemical reduction of CO_2_ to CO, but the nature of the Ni sites (Ni-N_2_C_2_, Ni-N_3_C_1_, Ni-N_4_) in Ni-SACs has not been determined experimentally. Here, we apply the recently developed grand canonical potential kinetics (GCP-K) formulation of quantum mechanics to predict the kinetics as a function of applied potential (U) to determine faradic efficiency, turn over frequency, and Tafel slope for CO and H_2_ production for all three sites. We predict an onset potential (at 10 mA cm^−2^) U_onset_ = −0.84 V (vs. RHE) for Ni-N_2_C_2_ site and U_onset_ = −0.92 V for Ni-N_3_C_1_ site in agreement with experiments, and U_onset_ = −1.03 V for Ni-N_4_. We predict that the highest current is for Ni-N_4_, leading to 700 mA cm^−2^ at U = −1.12 V. To help determine the actual sites in the experiments, we predict the XPS binding energy shift and CO vibrational frequency for each site.

## Abstract (web summary)

Single atom catalysts (SACs) are promising in electrocatalysis but challenging to characterize. Here, the authors apply a recently developed quantum mechanical grand canonical potential kinetics method to predict reaction mechanisms and rates for CO_2_ reduction at different sites of graphene-supported Ni-SACs.

## Introduction

Fossil fuels have been the primary source to produce energy for industrial applications and human activities for decades^1,2^, leading to high concentration of atmospheric CO_2_ that create serious environmental, ecological, and climate problems. It is now essential to find improved catalysts to convert CO_2_ to useful chemicals^3^. Indeed progress is being made in electrochemical transformation of CO_2_ into chemical fuels^4,5^, however, the extreme stability of CO_2_ makes it inert for chemical reactions in aqueous solution, demanding a large overpotential to overcome the high activation barrier. Moreover, the hydrogen evolution reaction (HER)  side reaction can easily dominate under the same conditions, diminishing efficiency^6–8^. Thus we want CO_2_ reduction catalysts with high faradic efficiency for CO but negligible HER faradic efficiency. The column 11 elements (IB group) (Cu, Ag, Au) and their alloys provide the best catalysts to date^9–14^, however, poor selectivity and stability limit their use in practical applications that which require high activity, excellent selectivity, and long-term stability. In recent years, single atom catalysts (SACs) have emerged as an effective strategy to create new electrocatalysts with maximal atomic efficiency and high selectivity for specific catalytic reactions^15–18^. Atomically dispersed metals on suitable substrates have demonstrated unique electronic properties with great potential for electrocatalytic CO_2_ reduction^19–21^. In particular, graphene, a single atomic layer of carbon, has proved to be an effective support. It has large surface area, high conductivity, high stability, and the capability to tune electronic properties by forming strong chemical bonds to guest atoms^22–24^. Several experimental studies have been published recently on the CO_2_ reduction reaction (CO_2_RR) for the nickel single atom catalysts (Ni–SACs) on graphene^20,21,25–29^, but the performance varies markedly perhaps because of differences in the number of carbon or nitrogen bonds to Ni.

Recent developments in quantum mechanics (QM) based methods provide new tools determine the reaction mechanisms for heterogeneous electrochemical reactions. Recently we extended the standard fixed electron QM to allow constant potential Grand Canonical QM (GC-QM) to describe the kinetics at fixed potential (U) for direct comparison to experiment^30^. In particular, we recently developed the grand canonical potential kinetics (GCP-K) method to combine fixed charge and fixed potential QM to allow the reaction barriers to change continuously as the applied potential is changed, leading directly to current versus potential relation (Tafel slope)^31^.

Here we report the application of GCP-K to predict the reaction mechanism and rates for CO_2_RR over Ni–SACs for the Ni–N_2_C_2_, Ni–N_3_C_1_, and Ni–N_4_ sites in graphene. We find that Ni–N_2_C_2_ leads to the lowest onset potential of −0.84 V (vs RHE) to achieve 10 mA cm^−2^ current density, leading to a Tafel slope of 52 mV dec^−1^ and a turn-over frequency (TOF) of 3903 h^−1^ per Ni site at neutral (pH 7) electrolyte conditions, showing best agreement with various experimental observations at lower overpotentials. We predict the onset potential for 10 mA cm^−2^ current density of −0.92 V for Ni–N_3_C_1_ and −1.03 V for Ni–N_4_ (which exhibits the highest saturation current for high applied potentials). We use quantum mechanics to predict the binding energy (BE) shift for the N and C 1*s* X-ray photoelectron spectroscopy (XPS) and the CO vibrational frequencies to help interpret the experimental nitrogen coordinations in Ni–SACs. We predict that the N 1*s* BE shift ranges from +1.18 to +0.96 eV for Ni–N_4_ and Ni–N_2_C_2_ respectively. The adsorbed CO intermediate vibrations range from 1985 cm^−1^ (perpendicular) at −1.0 V on Ni–N_2_C_2_ site to 1942 cm^−1^ in the *xz* plane at −1.25 V applied potential on the Ni–N_4_ site.

## Results

### Grand canonical potential kinetics formulation

Recently, we developed the GCP-K methodology to determine the kinetics for heterogenous electrochemistry as a function of applied potential while allowing the transition states to evolve continuously^31^. GCP-K uses a Legendre transformation to convert from fixed charge free energy, *F (n)*, to grand canonical, *G (n; U)*, allowing the thermodynamic free energy for heterogeneous electrochemical reactions to depend on the applied potential (U). The derivation starts with the general definition of the grand canonical potential^32–34^

1 $$G\left( {n;U} \right) = F\left( n \right) - ne(U_{{\mathrm{SHE}}} - U)$$

where *G* is the grand canonical free energy, which depends on the number of electron (*n*), applied potential (*U* vs SHE), total free energy (*F*) as a function of *n*, and electronic energy ($U_{{\mathrm{SHE}}} = \mu _{e,{\mathrm{SHE}}}$/*e*) at the standard hydrogen electrode (SHE) condition. The sign of *U* is defined as the potential used in experiments, i.e., *U* =  −0.5 V corresponds to −0.5 V vs SHE. We calculate how the number of electrons depends on the applied potential to obtain *G* (*n*;*U*) as a thermodynamic potential. To do this, we shift the Fermi level to correspond to the applied potential by changing the electronic band occupation, varying the number of electrons in the systems (Eq. 2)^35,36^. Finally, we obtain *GCP* (*U*) through minimizing *G* (*n*;*U*) according to (3)^37,38^

2 $$\frac{{{\mathrm{d}}G(n;U)}}{{{\mathrm{d}}n}} = 0\,{\mathrm{or}}\,\mu _e = \frac{{{\mathrm{d}}F(n)}}{{{\mathrm{d}}n}} = {\mathrm{e}}(U_{{\mathrm{SHE}}} - U)$$

3 $${\mathrm{GCP}}\,\left( U \right) = \min G\left( {n;U} \right) = \min \left\{ {F\left( n \right) - ne\left( {U_{{\mathrm{SHE}}} - U} \right)} \right\}$$

Approximating *F*(*n*) locally as quadratic and minimizing *G* (*n*;*U*) leads to a quadratic form in *GCP* (*U*). This quadratic dependence is consistent with the relevant empirical forms from other studies^39–41^. To obtain *GCP* (*U*) we fit a quadratic expansion of *F*(*n*):

4 $$F\left( n \right) = a(n - n_0)^2 + b(n - n_0) + c$$

where a, b, and c are determined from QM. Minimization of (4) (Supplementary Table 1–5) leads to Eq. (5).

5 $${\mathrm{GCP}}\left( U \right) = - \frac{1}{{4a}}(b - {\mathrm{\mu }}_{e,{\mathrm{SHE}}} + eU)^2 + c - n_0{\mathrm{\mu }}_{e,{\mathrm{SHE}}} + n_0eU$$

These parameters can be related to the physical quantities as follows,

First, the differential capacitance, $C_{{\mathrm{diff}}} = \frac{{\partial n}}{{\partial U}} = - \frac{1}{{2a}}{\mathrm{which}}\,{\mathrm{leads}}\,{\mathrm{to,}}\,{\mathrm{a}} = - \frac{1}{{2C_{{\mathrm{diff}}}}}$

Second, at the potential of zero charge, $n\left( {U_{{\mathrm{PZC}}}} \right) = n_0$, so we obtain

$$n\left( U \right) = - \frac{1}{e}\frac{{\partial {\mathrm{GCP}}\left( U \right)}}{{\partial U}} = n_0 - \frac{1}{{2ae}}\left( {b - {\mathrm{\mu }}_{e,{\mathrm{SHE}}} + eU} \right),$$

Thus, we can write, $b = {\mathrm{\mu }}_{e,{\mathrm{SHE}}} - eU_{{\mathrm{PZC}}}$

Finally, $F\left( {n = n_0} \right) = c$, when the system is neutral, the potential of zero charge (U_PZC_).

Putting these physical quantities into Eqs. (4) and (5), we write the grand canonical potential and free energy expressions (Supplementary Notes 1) as in Eqs. (6) and (7)

6 $$F\left( n \right) = - \frac{1}{{2C_{{\mathrm{diff}}}}}\left( {n - n_0} \right)^2 + \left( {{\mathrm{\mu }}_{e,{\mathrm{SHE}}} - eU_{{\mathrm{PZC}}}} \right)\left( {n - n_0} \right) + F_0$$

7 $${\mathrm{GCP}}\left( U \right) = \frac{{e^2C_{{\mathrm{diff}}}}}{2}\left( {U - U_{{\mathrm{PZC}}}} \right)^2 + n_0eU + F_0 - n_0{\mathrm{\mu }}_{e,{\mathrm{SHE}}}$$

where, $n_0$ is the number of electrons at zero net charge (total number of valence electron), $\mu _{e,{\mathrm{SHE}}}$ is the chemical potential of an electron vs standard hydrogen electrode (SHE), and *e* is the energy in eV. This quadratic form of free energy *F*(*n*) and grand canonical potential GCP (*U*) accounts for the change in capacitance as the potential changes.

Our procedure is first to carry out QM for a range of fixed charges using VASPsol solvation. Then we use the CANDLE solvation model implemented in jDFTx to obtain the free energies at the same constant charge. Then we recalculate the constant potential free energies using the Legendre transform. For stable intermediates the optimized geometry from VASPsol is used. For transition states we use the above procedure for each point along the climbing image nudged elastic band (CI-NEB) surface so that the transition state adjusts properly as the potential is changed.

### Structural model of Ni–SACs for CO_2_ reduction reaction

Experimental studies show that adding a Ni^2+^ containing metal solution to N-doped graphene oxide solution and then annealing at high temperature leads to electrocatalysts with Ni-nitrogen/carbon moieties embedded within the graphene matrix. The central Ni atom is coordinated to nitrogen/carbon(s) through strong covalent bonding, making this a single atom catalyst embedded within the graphene matrix. Each Ni may be bonded to 2 to 4 N’s atom along with 2–0 C’s, which we denote as Ni–N_2_C_2_, Ni–N_3_C_1_, or Ni–N_4_ (refs. ^16,27,42^). Our calculated formation energies show that Ni–N_4_ is energetically most favorable (−1.13 eV) while Ni–N_2_C_2_ structure (1.04 eV) is the least favorable. We examine here CO_2_RR considering all three possible structures and compare their catalytic performance.

**Fig. 1:**
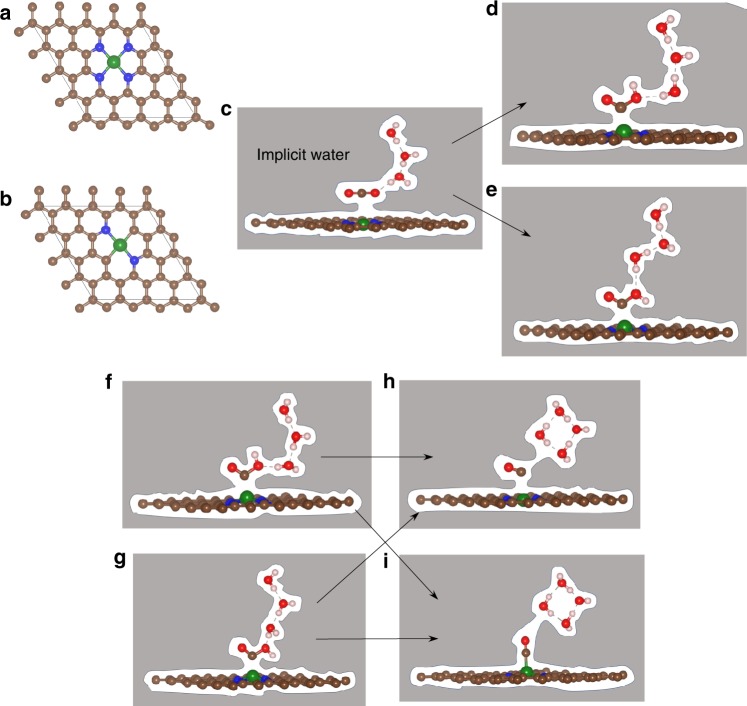
The CO_2_ to CO conversion process on graphene-supported nickel single site. **a**, **b** In all, 4 × 4 periodic Ni–SAC system containing Ni–N_4_ and Ni–N_2_C_2_ moieties as active sites for CO reduction reaction. **c** The optimized structure of physically adsorbed CO_2_ on Ni single site, is stabilized by three molecules of explicit water from solution. **d**, **e** The optimized structure of chemically adsorbed cis-COOH (H up) or trans-COOH (H down) respectively. The arrow sign represents the reaction direction where physically adsorbed CO_2_ molecule reacts with a neighboring water molecule to produces cis- or trans-COOH intermediates together with hydroxyl ion. The later is stabilized by two explicit water molecules in the solution. **f**, **g** Optimized adsorbed cis- and trans-COOH intermediates on Ni–SAC respectively. **h**, **i** Optimized structures of CO product binding on Ni–N_4_ and Ni–N_2_C_2_ site respectively. The arrow sign represents the reaction direction of chemisorbed COOH with water and produce CO and hydroxyl ion in solution. The CO binds on the Ni–N_2_C_2_ site perpendicularly representing a stronger attraction than on the Ni–N_4_ site. The whole calculation was done by using implicit solvation as in VASPsol and CANDLE solvation as implemented in the VASP and jDFTx code, respectively together with three explicit H_2_O to better describe the charge transfer and polarization. Supplementary Figs. 1 and 2 show that adding additional explicit waters leads to similar results, and supplementary Table 6 shows that reoptimizing the structures with jDFTx leads to negligible changes compared to the optimum structures in VASP. (Gray color to entire surface represents implicit solvation, brown: carbon, blue: nitrogen, green: nickel, red: oxygen, off white: hydrogen atom).

The reduction process of CO_2_ into CO involves two major steps, each involving a net transfer of one electron as the reactant is transformed to product (Fig. 1). We consider all three cases of Ni–N_4−*x*_C_*x*_ (*x* = 0–2) as possible configurations of Ni–SAC, embedded in a 4 × 4 periodic super cell of graphene, as shown in Fig. 1a, b. We examined the Eley-Rideal (ER) mechanism^43^ for CO_2_ reduction, where water from solution reacts with an adsorbed CO_2_ molecule to form *COOH and OH^−^ in solution. We start with one CO_2_ physisorbed on the single Ni sites including three explicit water molecules plus implicit solvation of this entire surface (Fig. 1c). As we apply a negative potential, the system builds up negative charge at the surface that induces linear CO_2_ to bend slightly on Ni–SAC. This bent CO_2_ facilitates a proton transfer from water to form the COOH intermediate, which binds strongly to the central Ni atom with Ni–C = 1.98–1.94 Å for various surface charges of 0–1.5 (a positive value represents excess electron compared to neutral, *n* − *n*_0_). This proton comes from a neighboring explicit water to form either cis-COOH (with the H pointing up) or trans-COOH (with the H pointing down). Simultaneously, product OH^−^ forms, stabilized by two other explicit water molecules (optimized) plus the implicit solvation, as shown in Fig. 1d, e respectively.

Figure 1f–i shows the second step of dehydroxylating the adsorbed COOH species by an explicit water to produce CO product plus H_2_O and OH^−^ (stabilized by water molecules and implicit solvent). The CO affinity on these Ni–SAC sites depends on the number of N’s bonded to the Ni, but none have a strong affinity for CO species (unlike Ni metal catalysts)^44,45^. Our calculations show that Ni-N_4_ has the least affinity for CO (Ni–CO bond distance ~2.53–2.27 Å), leading to tilted adsorbed CO (${\mathrm{\angle OCNi}} = 116^\circ$) (Fig. 1h) while Ni–N_2_C_2_ sites (Fig. 1i) show relatively stronger affinity toward CO (Ni–CO distance from 1.80 to 1.92 Å) and CO adsorbed almost linearly. This adsorbed CO desorbs from the Ni site along a straight pathway (${\mathrm{\angle OCNi}} = 176.5^\circ$) to slightly tilted (${\mathrm{\angle OCNi}} = 152^\circ$) depending on the applied potentials. The Ni–N_3_C_1_ site shows adsorption intermediate between Ni–N_2_C_2_ and Ni–N_4_ sites (Supplementary Data 1).

**Fig. 2:**
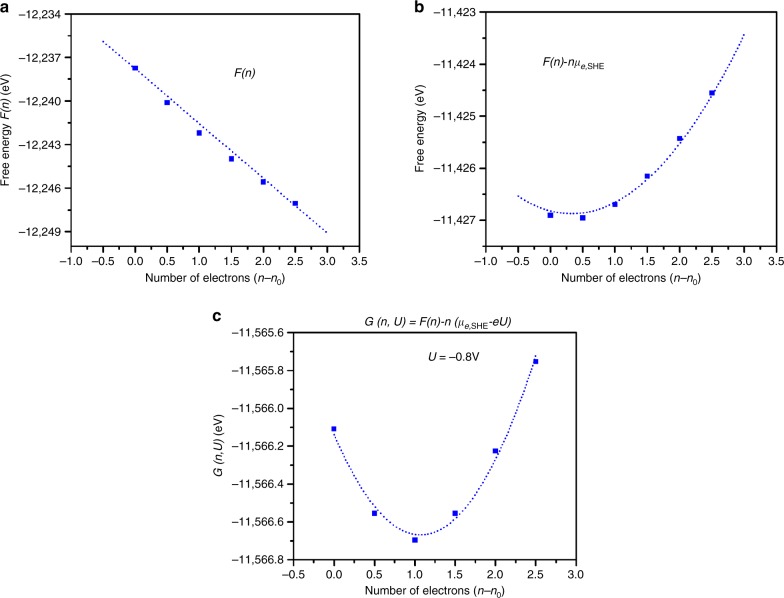
Behavior of free energy and grand canonical potential as a function of the number of electrons. **a** The free energies as a function of number of electrons show a nearly linear relationship. **b** The relation between free energy and number of electrons becomes quadratic when the energy contribution of each electron at SHE are subtracted from total free energy. The quadratic dependence allows the optimum number of electrons within the system. **c** Minimization of free energy as a function of number of electron when an external potential is applied to the system. The free energy minimum is shifted toward higher electron numbers when voltage applied, ensuring that the reaction progresses in the forward direction. The blue dots represent DFT energies while the dash curve refers polynomial 2nd order fitting. Our calculated rates use cubic splines to fit the points.

The quadratic behavior of the grand canonical potential was examined thoroughly for the whole process. The dependence for the trans-COOH reaction on applied potential is shown in Fig. 2. Figure 2a shows that the free energy, *F(n)* obtained from the CANDLE implicit solvation method, correlates linearly with system charge. However, subtracting the free energy contribution of each electron at SHE leads to the quadratic dependence shown in Fig. 2b, according to Eq. (6) leading to Eq. (8),

8 $$F\left( n \right) - \mu _{e,{\mathrm{SHE}}}n = - \frac{1}{{2C_{{\mathrm{diff}}}}}\left( {n - n_0} \right)^2 - \mu _{e,{\mathrm{SHE}}}n_0 - eU_{{\mathrm{PZC}}}\left( {n - n_0} \right) + F_0$$

Figure 2b shows that the optimum charge is slightly negative. Thus, at zero potential, the intermediate has a little more extra charge than the neutral. In electrochemical conditions, applying a negative potential, *U*, shifts the free energy minimum of *G (n, U*) by *neU* in Eq. (8). Figure 2c shows that this shifts the minimum of *G (n, U*) toward higher charge (the case of *U* = −0.8 V is shown Fig. 2c), which drives the reaction toward the product containing more charge.

### Transition states change as a function of applied potential

We first carried out constant charge DFT and then transformed the free energies to obtain GCP (*U*), leading to transition states (TS) exactly equivalent with the results from constant potential calculations (as shown in our earlier work using the minimax theorem)^31,46^. The free energy of the transition state, *F*_TS_(*n*) obtained from the fixed charge (*n*) calculation is transformed into the grand canonical potential GCP_TS,*n*_ (*U*) via a Legendre transformation. Since the transition state is the highest barrier along the minimum energy path (MEP), the TS geometry changes with applied potential, *U*.

**Fig. 3:**
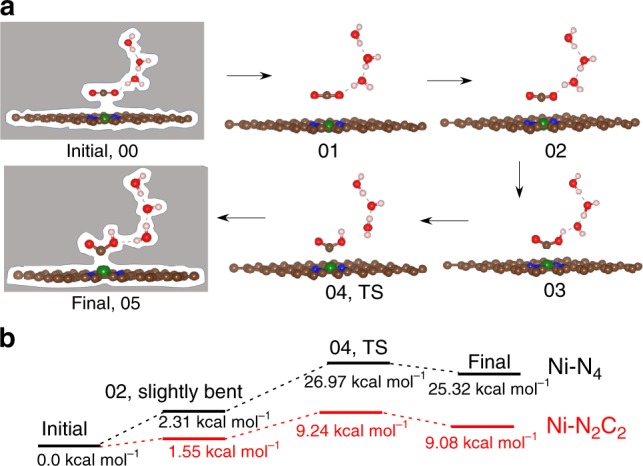
Reaction pathways and barriers for CO_2_ to cis-COOH conversion. **a** The minimum energy path calculation using the CI-NEB method with implicit solvation. We consider total six images (00–05) for this process. The initial linear CO_2_ interacts with neighboring water molecule to form a slightly bent structure (image 02). Later this bent CO_2_ takes a proton from water to produce cis-COOH product (05 image) via the transition state (TS) at image 04. The arrow shows the forward reaction. **b** Reaction energies and TS barrier for the protonation step for CO_2_ reduction on the Ni–N_4_ and Ni–N_2_C_2_ sites at −0.8 V vs RHE applied potential. The proton transfer occurs most easily on Ni–N_2_C_2_, followed by Ni–N_3_C_1_ and Ni–N_4_ site. Note that in these calculations the charge of the system changes continuously as the H moves from water to OCO to form OCOH* and OH^−^. There is not a separate electron transfer step as in the proton coupled electron transfer (PCET) surface hopping picture. (Gray color: entire surface represents implicit solvation along the whole reaction pathways, brown: carbon, blue: nitrogen, green: nickel, red: oxygen, off white: hydrogen atom).

**Fig. 4:**
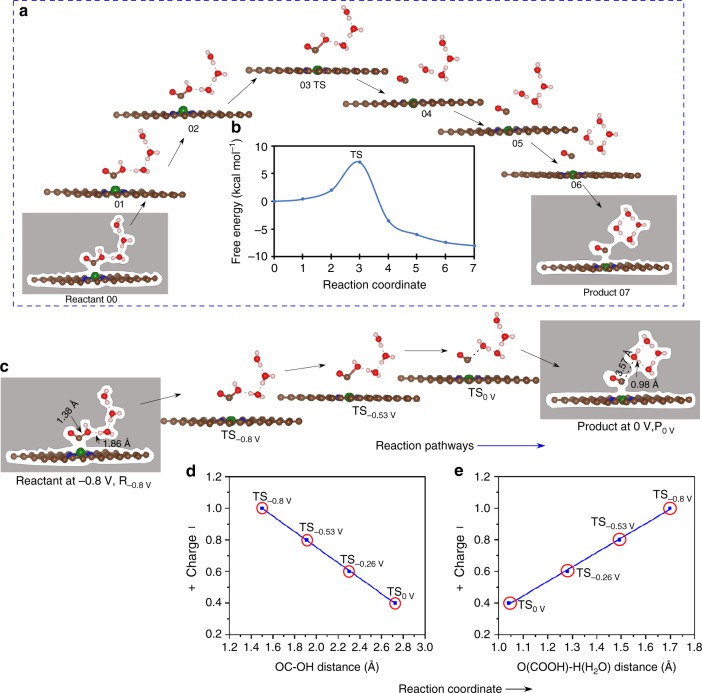
COOH to CO reaction pathways and transition states at different potential. **a** The minimum energy path (MEP) for cis-COOH to CO conversion at −0.53 V applied potential (0.8 charge) on Ni–N_4_ site. We use a total of eight images (00–07) for this conversion process. During the reaction of cis-COOH with water, the OC–OH bond length increases from 1.38 to 1.86 Å with a transition state (TS) at image 03. Eventually it forms CO by breaking OC–OH bond. The arrow indicates the forward direction of the reaction via MEP. **b** The free energy barrier for the cis-COOH to CO at −0.53 V applied potential via MEP. Image 03 (TS) shows the maximum energy barrier of 7.02 kcal mol^−1^ at −0.53 V applied potential for the conversion of cis-COOH to CO on Ni–N_4_ site. **c** The transition state (TS) between reactant cis-COOH and product CO changes with applied potential. Staring from product, at zero applied potential the product (P_0V_) CO is 3.57 Å (OC–OH) distance away from neighboring water while the O–H bond distance in water is 0.98 Å. The transition state at zero (TS_0V_) applied potential shows a geometry similar to the product. At −0.53 V applied potential, the OC–OH distance at the transition state (TS_−0.53V_) decreases while the O-H bond increases. With a more negative applied potential of −0.8 V, the OC–OH bond of the transition state (TS_−0.8V_) becomes very early, closer to the reactant at same potential. The reactant at −0.8 V (R_−0.8V_) has OC–OH = 1.38 Å and O–H = 1.86 Å. The arrow indicates the reactant to product direction. **d** The relation of distance between OC–OH to the fractional charge in the TS as a function of applied potential. **e** The change of distance between O-H and the fractional charge in the TS as a function of applied potential. The red circle represents the TS at different applied potentials. (Gray color: entire surface represents implicit solvation along the whole reaction pathways, brown: carbon, blue: nitrogen, green: nickel, red: oxygen, off white: hydrogen atom).

Figure 3 shows how the CO_2_ to OCOH reaction barrier depends on the applied potential for the three types of Ni sites along the minimum energy path and transition states at *U* = −0.8 V vs RHE. The conversion of linear CO_2_ through bent CO_2_ to COOH is endothermic. The slightly bent state (image 02) is followed by the transfer of a H from HOH to form OCOH plus an OH^−^ stabilized by the two H_2_O (images 03–04). The transition state (image 04) has a geometry similar to the *COOH intermediate but with a slightly higher energy (1.65 kcal mol^−1^) arising from reorientation of the bottom water molecule (Fig. 3a). This protonation step has an energy barrier shown in Fig. 3b. For Ni–N_2_C_2_, the linear CO_2_ (initial state) (${\mathrm{\angle}} {\mathrm{OCO}} = 178.34^\circ$), first becomes slightly bent (${\mathrm{\angle}} {\mathrm{OCO}} = 167^\circ$) at the 02 image leading to a low energy barrier (1.55 kcal mol^−1^ at *U* = −0.8 V), indicating fast decoupled electron transfer followed by proton transfer with higher energy barrier (9.24 kcal mol^−1^) at image 04. For Ni–N_3_C_1_, we find a 1.97 kcal mol^−1^ energy barrier to form slightly bent CO_2_ and a barrier of 17.72 kcal mol^−1^ to form OCOH. Finally, for Ni–N_4_ we find a 2.31 kcal mol^−1^ energy barrier to form slightly bent CO_2_ and a barrier of 26.97 kcal mol^−1^ to form OCOH at −0.8 V potential. The reaction pathways and energies for the conversion of linear CO_2_ to trans-COOH are shown in Supplementary Fig. 3. In contrast to step 1, the reaction pathway for conversion of cis-COOH to CO clearly involves a sharp TS and lower energy barrier at constant applied potential as shown in Fig. 4a, b. The reaction pathway shows the bond breaking between OC–OH step (image 3) has the highest energy barrier. Among all studied sites, Ni–N_3_C_1_ shows the highest barrier of 10.01 kcal mol^−1^ while Ni–N_4_ shows the lowest barrier of 7.02 kcal mol^−1^ at *U* = −0.53 V applied potential (Fig. 4b). The pathways with free energy for the formation of CO from trans-OCOH is shown in Supplementary Fig. 4.

Figure 4c–e illustrates the geometric change of the TS as a function of applied potential along the reaction path. Along the reaction path (Fig. 4a), we show two spatial coordinates: (1) distance between OC–OH in the COOH TS, which changes from 1.46 Å to 2.77 Å as *U* = −0.8 changes to 0 V (Fig. 4d) and (2) the distance between the new OH bond to the OH of COOH and the old OH bond of the H_2_O molecule donating the proton changes from 1.71 Å to 1.04 Å as *U* =  −0.8 V changes to 0 V (Fig. 4e).

**Fig. 5:**
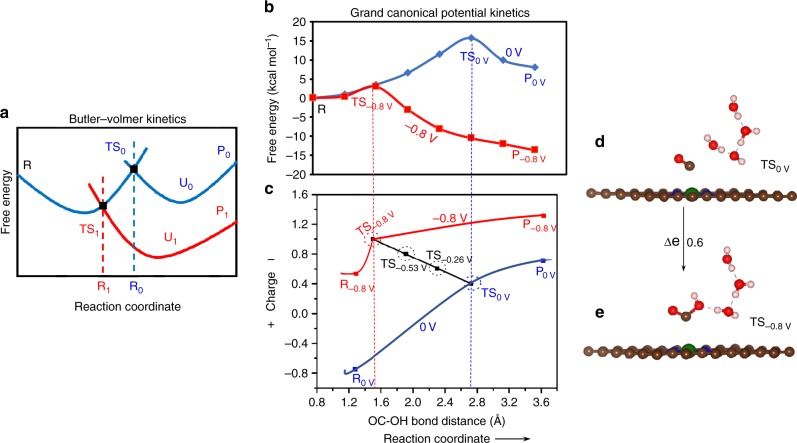
Schematic and quantitative description of the reaction kinetics. **a** The traditional Butler–Volmer reaction kinetics. Starting from product (blue curves), at zero applied potential, (*U*_0_) the reaction between reactant, R and product, P_0_ shows a transition state (TS_0_) with R_0_ spatial distance along the reaction path. The transition state (TS_1_) shifts toward the reactant with lower in free energy gives R_1_ spatial distance at applied potential U_1_ between reactant R and product P_1_ (red curves). The product forms with a single electron transfer from electrode to product, leading an electron transfer jump along the reaction pathway. **b**, **c** Grand canonical potential kinetics methodology, illustrating the relationship between the TS geometry and charge as the reaction changes continuously with applied potential, leading to a continuous potential dependent reaction path. The blue curve represents the reaction pathways for cis-COOH to CO conversion on Ni–N_4_ site at zero (0 V) potential while the red curve is for −0.8 V applied potential. At 0 V, the reaction between reactant, R or R_0V_ and product, P_0V_ produces a transition state (TS_0_) with OC–OH = 2.77 Å (blue dotted line) and an energy barrier of 16.89 kcal mol^−1^. At −0.8 V applied potential the energy barrier for the transition state TS_−0.8V_ decreases to 2.98 kcal mol^−1^ with OC–OH = 1.46 Å (red dotted line). The black line connecting the transition states at 0 V (TS_0_) and −0.8 V (TS_−0.8V_) shows the linear continuous relation between the applied potential and species charge within the system. The dotted circles indicate the transition states at different applied potentials. **d**, **e** The geometry of the transition state at zero (TS_0_) and −0.8 V (TS_−0.8V_) applied potential, resulting a charge difference of 0.6 *e*. The arrow represents the TS direction on applied potential. (brown: carbon, blue: nitrogen, green: nickel, red: oxygen, off white: hydrogen atom).

Our results show that the TS at zero applied potential is very similar to the product but as *U* becomes more negative, the TS moves toward the reactant, becoming similar to the reactant at *U* = −0.8 V (Fig. 4c). As the potential becomes more negative, the reaction barrier decreases making the path more favorable to convert reactants into products. Thus, we find the OC–OH distance decrease uniformly with *U* (Fig. 4d) and the COOH–H_2_O distance increases uniformly (Fig. 4e). The charges associated with the adsorbed species change linearly with *U*, leading to a smooth reaction pathway along the reaction coordinate. At zero potential, the transformation begins with a charge of 0.4 on the Ni–SACs, which increases to 1.0 as the applied potential is increased to −0.8 V. To show the linear relationship between applied potential and charges on the species more quantitatively, we compare this reaction path for the GCP-K model with the conventional Butler–Volmer PCET kinetics in Fig. 5. This shows that at potential *U*_*0*_, the transition state, TS_0_ (Fig. 5a) corresponds to the spatial geometry R_0_ but as the applied potential changes to *U*_1_ the transition state (TS_1_) moves leftward toward reactant reaching to R_1_ (refs. ^47,48^). In this grand canonical potential kinetics (GCP-K) description, the fractional charges change continuously along the reaction coordinates as the applied potential is changed, as shown in Fig. 5b, c. Initially, at zero voltage the reaction barrier is 16.89 kcal mol^−1^, the TS has a slightly negative charge (0.4 *e*) and the OC–OH distance is about 2.77 Å, very similar to the product (Fig. 4c). But as the applied potential is changed to −0.8 V, the barrier decreases continuously to 2.98 kcal mol^−1^ while the TS shifts continuously toward the reactant with a short (1.46 Å) OC–OH distance at a more negative charge of 1.0 *e*. The charge difference between the transition states at 0 V (TS_0V_) and −0.8 V (TS_−0.8_) is 0.6 *e* as shown in Fig. 5d, e. Thus, we describe the charge transfer as a potential dependent continuous process during the electrochemical reaction as the intermediates adsorb on the electrode surface and react along the reaction path leading to a smooth path for the reaction. Similarly, we observe a continuous change of the transition state (TS) with applied potential for the trans-COOH to CO conversion reaction for the Ni–N_4_ site (Supplementary Fig. 5). We also observe similar behavior for the reaction at the Ni–N_2_C_2_ and Ni–N_3_C_1_ sites. As shown above, the conversion of CO_2_ to COOH leads to a lower energy barrier for Ni–N_2_C_2_ and Ni–N_3_C_1_ sites than for Ni–N_4_. This makes the 2nd step reaction slightly less favorable for Ni–N_2_C_2_ and Ni–N_3_C_1_ sites. The conversion of cis-COOH and trans-COOH into CO on Ni–N_3_C_1_ and Ni–N_2_C_2_ involve higher energy barriers than on Ni–N_4_ (Supplementary Figs. 6–8).

### The hydrogen evolution reaction on Ni–SACs

**Fig. 6:**
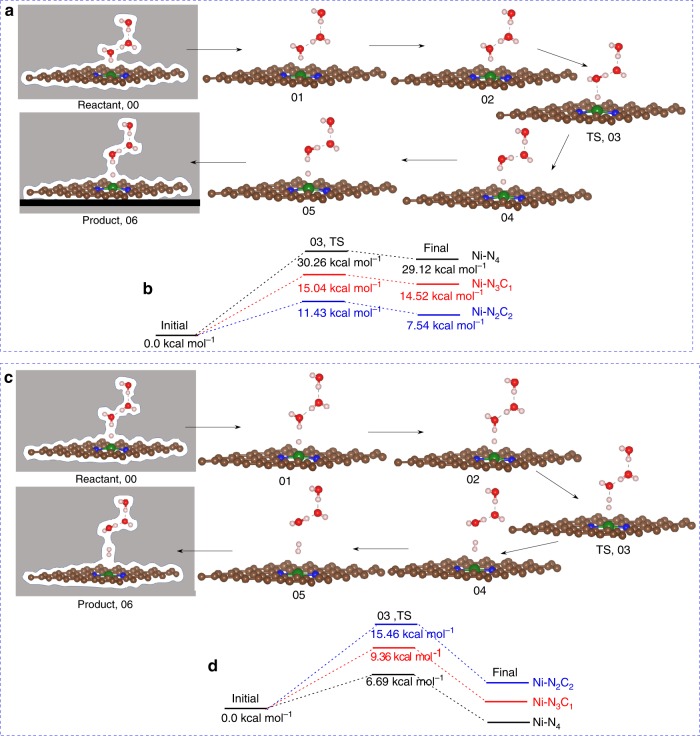
Reaction pathways for hydrogen evolution reaction (HER) on nickel single site. **a** The minimum energy reaction path for proton adsorption (Volmer step) from solution to the nickel single site at −0.8 V applied potential. We use total seven images (00–06) for this adsorption step. The hydrogen near the single nickel at point 00 gradually moves toward the nickel site leading to a transition state (TS) at 03 before converting into the adsorbed final state at 06. **b** The adsorption free energies and TS barrier for the Volmer step of HER on different nickel sites at −0.8 V vs RHE applied potential. Among all sites, Ni–C_2_N_2_ shows a lower activation barrier for proton transfer at the active site. **c** The Heyrovsky reaction path for hydrogen gas evolution from the nickel surface at −0.8 V applied potential. We use seven images (00–06) to describe the H_2_ desorption step. The adsorbed hydrogen at point 00 reacts with a proton from solution to form H_2_ gas at 06, leading to a transition state at point 03. **d** Desorption reaction barrier for the Heyrovsky step of HER on different nickel sites at −0.8 V vs RHE applied potential. The H_2_ evolution is difficult from Ni–C_2_N_2_ site because its strong adsorption energy for the Volmer step. The arrow indicates the forward reaction pathways for adsorption and desorption steps, respectively. (Gray color: entire surface represents implicit solvation along the whole reaction pathways, brown: carbon, blue: nitrogen, green: nickel, red: oxygen, off white: hydrogen atom).

The hydrogen evolution reaction (HER) competes with the CO_2_RR, decreasing the faradic efficiency (FE) for CO evolution. HER can take place either by reducing protons ($2{\mathrm{H}}^ + + 2{\mathrm{e}}^ - \to {\mathrm{H}}_2$) or by reduction of water $(2{\mathrm{H}}_2{\mathrm{O}} + 2{\mathrm{e}}^ - \to {\mathrm{H}}_2 + 2{\mathrm{OH}}^ - )$ from aqueous solution via the Volmer-Tafel/Heyrovsky reaction. Thus, we must examine the comparative activity of HER and CO_2_RR on the same catalysts and electrolyte conditions. Since our electrolytic environment is neutral (pH 7), we considered the HER via water reduction. Therefore, there are two steps: In step 1 shows hydrogen from solvent water gradually moves toward Ni surface and finally absorbs onto the Ni site via the Volmer reaction, producing OH^−^ solvated by the explicit H_2_O molecules (Fig. 6a) and step 2 involves the adsorbed hydrogen slowly combining with a second hydrogen from solution H_2_O to finally evolves as H_2_ gas via the Heyrovsky reaction (Fig. 6c). Figure 6b, d show that HER depends strongly on the number of N bonded to the Ni. Thus, at pH 7 and *U* = −0.8 V applied potential we find that the hydrogen adsorption on Ni–N_2_C_2_ site from solvent water involves a transition state (TS) with an energy barrier of 11.43 kcal mol^−1^(Fig. 6b), while it requires relatively higher energy of 15.46 kcal mol^−1^ to combine with a second hydrogen from water in solution to evolves as H_2_ gas via Heyrovsky step (Fig. 6d). On the other hand, the calculated reaction barrier for the Volmer step to put H* on the surface is 15.04 kcal mol^−1^ and the Heyrovsky step of forming H_2_, it is 9.36 kcal mol^−1^ on Ni–N_3_C_1_ sites. Here the H* is adsorbed on the bridge sites of the Ni–C bond (Supplementary Figs. 9–10). The bond distances of C–H and Ni–H in the bridge site position are about 1.14 and 1.80 Å, respectively. The Ni–N_4_ sites are least favorable for HER. The calculated reaction barrier for the Volmer step is 30.26 kcal mol^−1^, while the Heyrovsky step has a barrier of 6.69 kcal mol^−1^ at pH 7 and applied potential −0.8 V conditions (Supplementary Figs. 9–12). The Ni–N_4_ sites are preferred to minimize HER.

### Reaction mechanism for CO_2_ reduction

**Fig. 7:**
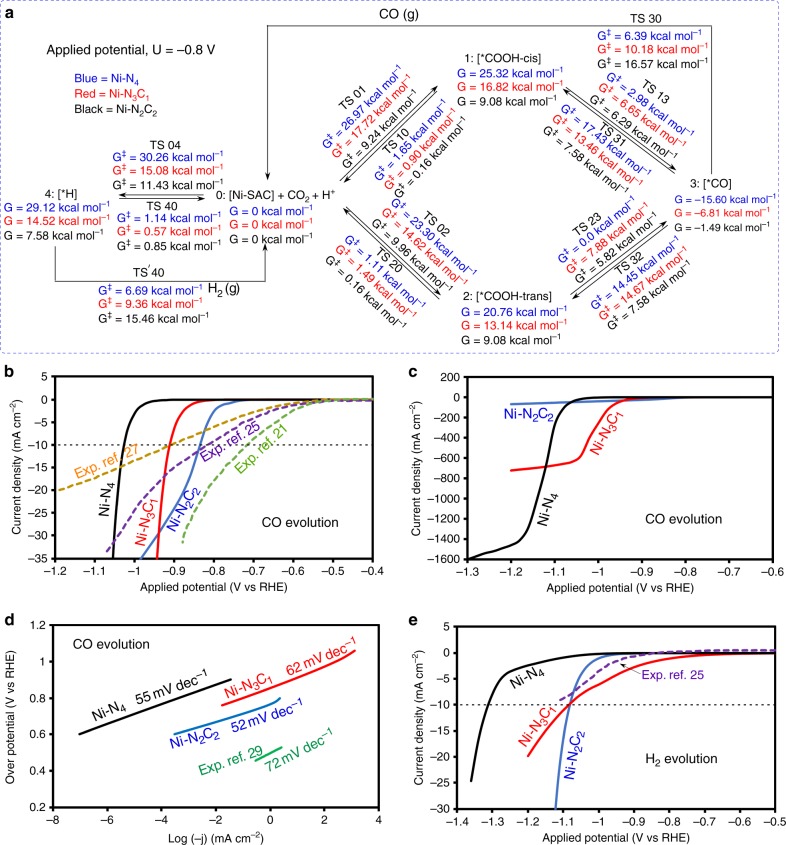
QM derived free energies and predicted reaction kinetics. **a** Schematic representation of free energies at 298 K and pH 7 for −0.8 V applied potential. This summarizes all reaction intermediates (0–4) and transition state (TS) free energies involved in the reduction of CO_2_ on nickel single site at −0.8 V constant applied potential. (Blue: Ni–N_4_, Red: Ni–N_3_C_1_ and Black: Ni–N_2_C_2_ surface). **b** Calculated partial current densities for CO evolution during CO_2_ reduction on Ni–N_2_C_2_ (blue curve), Ni–N_3_C_1_ (red curve) and Ni–N_4_ (black curve) along with experimental data from refs. ^21,25,27^ (dotted curve) for comparison (equivalent number of nickel atoms). **c** Large partial current densities for CO evolution during CO_2_ reduction on Ni–N_2_C_2_ (blue curve), Ni–N_3_C_1_ (red curve) and Ni–N_4_ (black curve). **d** Tafel slopes calculated from the I–V curves for CO evolution on Ni–N_2_C_2_ (blue line), Ni–N_3_C_1_ (red line) and Ni–N_4_ (black line) during CO_2_ reduction, showing good agreement with the Tafel slope from experiment (green line). **e** Calculated partial H_2_ current densities as a function of applied potential during CO_2_ reduction on Ni–N_2_C_2_ (blue curve), Ni–N_3_C_1_ (red curve) and Ni–N_4_ (black curve) along with experimental data from ref. ^25^ (dotted curve) for comparison (equivalent number of nickel atoms), at pH 7 and 298 K.

We calculated the CO_2_ reduction reaction at neutral pH condition on Ni–SACs with three different Ni sites configurations: Ni–N_2_C_2_, Ni–N_3_C_1_, and Ni–N_4_ embedded in graphene. The reference fermi energy of the electron is shifted by pH × 0.059 eV to put the applied potential in terms of RHE. We summarize the free energy activation barriers and reaction free energies in Fig. 7a obtained using grand canonical potential GCP (*U*) kinetics for all possible intermediates, transition states, and products during CO_2_ reduction at pH 7 and −0.8 V vs RHE applied potential.

Overall, CO_2_ reduction on Ni–N_2_C_2_ site via the cis pathway is most favorable at *U* = −0.84 V, the onset potential for 10 mA cm^−2^ current density. But in the last step of forming CO, the bond to Ni is too strong, limiting the maximum current at more negative potentials. The CO_2_ conversion to cis-COOH reaction is energetically most favorable on Ni–N_2_C_2_ sites. In the first step the applied potential adds negative charge to CO_2_, causing it to bend. Next it reacts with a proton from a solution H_2_O to form cis-COOH via TS01 with a barrier of 9.24 kcal mol^−1.^ This cis-COOH can convert back into CO_2_ with a small barrier (TS10) of 0.16 kcal mol^−1^ or it can abstract a second hydrogen from solution to form CO plus H_2_O and OH^−^ with a barrier (TS13) of 6.29 kcal mol^−1^. This CO is strongly adsorbed on the central Ni surface; to evolve as CO gas requires a barrier (TS30) of 16.57 kcal mol^−1^. This desorption frees up the binding site for further reaction or it can form cis-COOH again by the back reaction with a barrier (TS31) of 7.58 kcal mol^−1^. On the other hand, linear CO_2_ is converted into trans-COOH on Ni–N_2_C_2_ sites with a barrier (TS02**)** of 9.96 kcal mol^−1^, slightly higher than cis although the reverse process has a same barrier (TS20) of 0.16 kcal mol^−1^ or it can react with another hydrogen from solution to form adsorbed CO with slightly lower barrier (TS23) of 5.82 kcal mol^−1^. This adsorbed CO is finally evolved into solution as CO gas with the same barrier (TS30) as cis, 16.57 kcal mol^−1^, leaving an empty catalyst surface or it can convert into trans-COOH by back reaction with high barrier (TS32) of 7.58 kcal mol^−1^.

Although Ni–N_2_C_2_ sites initiate the CO production at the lowest *U*, Ni–N_3_C_1_ dominates in the potential range of *U* = −0.92 V to −1.12 V, where the maximum current is limited due to strong bonding of CO to the Ni site. The conversion of CO_2_ to cis-COOH on the Ni–N_3_C_1_ site leads to a barrier (TS01) of 17.72 kcal mol^−1^, which is more uphill than Ni–N_2_C_2_ site. This reaction can be reversed with a very small barrier (TS10) of 0.90 kcal mol^−1^ or it can abstract a second hydrogen from solution and convert into CO with a forward barrier (TS13) of 6.65 kcal mol^−1^. This adsorbed CO has intermediate affinity on Ni and can evolve as CO gas with a barrier (TS30) of 10.18 kcal mol^−1^, leaving the Ni site vacant for further reactions or it can form cis-COOH again by the reverse reaction with a barrier (TS31) of 13.46 kcal mol^−1^. The conversion involving the trans-COOH intermediate on N–N_3_C_1_ sites is slightly easier than for the cis form. Here, the CO_2_ is converted into trans-COOH with a barrier (TS02**)** of 14.62 kcal mol^−1^, the reverse reaction to produce CO_2_ has a small barrier (TS20) of 1.49 kcal mol^−1^ or it can react with another hydrogen from solution to form adsorbed CO plus H_2_O and OH^−^ with a relatively higher barrier (TS23) of 7.88 kcal mol^−1^ than for cis case. The adsorbed CO can evolve as CO gas with a barrier (TS30) of 10.18 kcal mol^−1^, leaving the active site available for next reaction or it can convert into the trans-COOH by the reverse reaction with a high barrier (TS32) of 14.67 kcal mol^−1^.

Ni–N_4_ sites produce the maximum current for CO production with higher faradic efficiency, but at higher potentials. The trans pathway provides a lower energy barrier than cis for both steps of CO production. The CO_2_ conversion to cis-COOH (Supplementary Note 2) is energetically uphill by 26.97 kcal mol^−1^ via the TS01 step on Ni–N_4_ site, much higher than the other Ni sites. The cis-COOH can convert back into CO_2_ with a barrier (TS10) of 1.65 kcal mol^−1^ or it can abstract a second hydrogen from solution to form adsorbed CO at a barrier (TS13) of 2.98 kcal mol^−1^. This CO is very weakly adsorbed on the Ni site and evolves as CO gas with a barrier (TS30) of 6.39 kcal mol^−1^, leaving free the binding site for further reaction or it can form cis-COOH again by back reaction with a barrier (TS31) of 17.43 kcal mol^−1^. The CO_2_ to CO conversion along the trans path has a lower energy barrier because the trans-COOH intermediate is more stable than cis-COOH. The CO_2_ is converted into trans-COOH with a lower barrier (TS02**)** of 23.30 kcal mol^−1^. The reverse process has low barrier (TS20) of 1.11 kcal mol^−1^ or it can react with another hydrogen from solution to form adsorbed CO with no barrier (TS23). This weakly adsorbed CO is finally evolved from solution as CO gas with the same barrier (TS30), leaving an empty catalysts surface or it can convert into trans-COOH by back reaction with high barrier (TS32) of 14.45 kcal mol^−1^.

### Overall kinetics and comparison with experiments

The Free energies of all reaction intermediates and transition states were calculated as a function of applied potential using our quadratic transformation of the grand canonical potential. Since, the reaction rates depend directly on the applied potential, we obtain the rates from the free energy change to the TS using the Eyring rate equation. Finally, the overall reaction rate and species concentration are calculated using a microkinetic model. The rate equations for each species are as follows below:

$$\begin{array}{l}\frac{{{\mathrm{d}}x_0}}{{{\mathrm{dt}}}} = - \left( {k_{01} + k_{02} + k_{04}} \right)x_0 + k_{10}x_1 + k_{20}x_2 + k_{30}x_3 + (k_{40} + k{\prime}_{40})x_4\\ \frac{{{\mathrm{d}}x_1}}{{{\mathrm{dt}}}} = k_{01}x_0 - \left( {k_{10} + k_{13}} \right)x_1 + k_{31}x_3\\ \frac{{{\mathrm{d}}x_2}}{{dt}} = k_{02}x_0 - \left( {k_{20} + k_{23}} \right)x_2 + k_{32}x_3\\ \frac{{{\mathrm{d}}x_3}}{{{\mathrm{dt}}}} = k_{13}x_1 + k_{23}x_2 - \left( {k_{31} + k_{32} + k_{30}} \right)x_3\\ \frac{{{\mathrm{d}}x_4}}{{{\mathrm{dt}}}} = k_{04}x_0 - (k_{40} + k{\prime}_{40})x_4\end{array}$$

and, the Eyring rate equation for rate constant:

$$k_{ij}\left( U \right) = \frac{{k_BT}}{h}{\mathrm{exp}}\left( {\frac{{ - \Delta G_{ij}^\ddagger \left( U \right)}}{{k_BT}}} \right)$$

where, $x_i$ is the concentration of each species, $k_{ij}(U)$ is the potential dependent rate constant, $k_B$ is the Boltzmann’s constant, and *h* is the Planck’s constant. Here we apply the constraint ${\mathrm{\Sigma }}_ix_i = 1$. Solving for the rate at the onset potential for each case, we obtain the concentration of each species as summarized in Table 1.

**Table 1:** Species concentration corresponding to the onset potential of each site at 298 K and pH = 7.

Species	Conc. (Ni–N_2_C_2_)	Conc. (Ni–N_3_C_1_)	Conc. (Ni–N_4_)
[Ni–SAC] (*x*_0_)	0.88736	0.92943	0.99165
[*cis-COOH] (*x*_1_)	8.769 × 10^−05^	4.051 × 10^−04^	6.471 × 10^−08^
[*trans-COOH] (*x*_2_)	3.221 × 10^−06^	1.199 × 10^−04^	1.111 × 10^−06^
[*CO] (*x*_3_)	0.11259	0.07003	0.00835
[*H] (*x*_4_)	2.5616 × 10^−05^	5.087 × 10^−05^	1.649 × 10^−08^
Onset potential, *U*_onset_ for 10 mA cm^−2^	−0.84 V	−0.92 V	−1.03 V

The sum of all species concentrations is 1.

Solving these equations to obtain the kinetics as a function of applied potential leads to the current density as a function of applied potential shown in Fig. 7b–e for the CO_2_ reduction reaction. Depending on each reaction rate, the concentrations change as a function of the applied potential. Figure 7b, c shows the CO partial current densities as a function of applied potential for the Ni–N_2_C_2_, Ni–N_3_C_1_ and Ni–N_4_ catalysts. The I–V curves show that CO_2_RR starts first from Ni–N_2_C_2_ sites at −0.7 V with *U*_onset_ (10 mA cm^−2^) = −0.84 V. At *U* = −0.84 V, we calculate the CO faradic efficiency (FE) to be 98% with a turn-over-frequency (TOF) of 3903 h^−1^ per site. We calculate the Tafel slope for Ni–N_2_C_2_ to be 52 mV dec^−1^ (Fig. 7d), which is dominated by fast electron transfer followed by a slow protonation step converting CO_2_ to COOH as the rate limiting step (RDS). We find that for *U* < −0.9 V the current tends to saturate with a very large Tafel slope due to the strong CO bond to the Ni site. For *U* < −0.93 V, Ni–N_3_C_1_ becomes more active than Ni–N_2_C_2_ site, which are blocked by strong CO binding (Fig. 7c). For the Ni–N_3_C_1_ site, we obtain *U*_onset_ = −0.92 V, FE = 78% and TOF = 2940 h^−1^ with a maximum Tafel slope of 62 mV dec^−1^. The Tafel slope drastically increases for *U* < −1.06 V due to strong CO bonding to Ni site. Then as *U* < −1.05 V, Ni–N_4_ dominates CO production. For Ni–N_4,_ we find *U*_onset_  = −1.03 V with a high FE = 99%, and TOF = 3944 h^−1^ with a Tafel slope of 55 mV dec^−1^. Here the binding of CO to the Ni is weaker, so that very high currents are predicted (this may be limited by transport and other issues). Overall, the Ni–N_2_C_2_ site dominates CO production for *U* ~ −0.85 V, with Ni–N_3_C_1_ dominating by −0.95 V, and Ni–N_4_ dominating by −1.12 V. For comparison, Fig. 7b shows the experimental data for CO_2_RR from three papers, normalized (Supplementary Note 3) to have the same active sites concentrations^21,25,27^. The recent experimental papers lead to an onset potential at 10 mA cm^−2^ of −0.7, −0.84, and −0.93 V in reasonable agreement with our predictions for the Ni–N_2_C_2_ site (*U*_onset_ = −0.84 V) and Ni–N_3_C_1_ site (*U*_onset_ = −0.92 V)^20,25,27,28,49^. Thus, we conclude that experimental Ni–SACs may have all three sites in different proportions contributing to the overall CO_2_ reduction performance.

The I–V curves for HER are shown in Fig. 7e. Most notable is that HER for Ni–N_3_C_1_ site starts at *U* = − 0.72 V, reaches 5 mA cm^−2^ at −0.96 V, and 10 mA cm^−2^ at −1.09 V. In contrast HER for Ni–N_2_C_2_ and Ni–N_4_ do not start until *U* < −0.98 V. This suggests that to reduce HER the synthesis should attempt to minimize Ni–N_3_C_1_ sites and use applied potentials less negative than *U* = −1.0 V where CO performance is reasonably high. The Tafel slopes obtained for HER on Ni–N_2_C_2_ sites is 100 mV dec^−1^ and for Ni–N_3_C_1_ it is 153 mV dec^−1^ (Supplementary Fig. 13). This can be compared to the experimental value of 140 mV dec^−1^^25^. For Ni–N_4_ site, HER is very slow, leading to *U*_onset_ = −1.31 V for 10 mA cm^−2^, with a Tafel slope 84 mV dec^−1^. Thus Ni–N_4_ is the best overall performer with a current of 40 mA cm^−2^ at *U* = −1.05 V with CO FE ~ 100%.

### Comparison of QM based descriptors with experiments

In order to find the synthesis conditions that maximize production of the desired sites, say Ni–N_4_ it would be useful to have descriptors indicating which sites dominate prior to experimentally inserting into a cell. The CO stretching vibration is prominent in the FT-IR, occurring experimentally at 1900–2060 cm^−1^ on Ni single sites^50^. We calculated the vibration wavenumbers for adsorbed CO on different Ni-sites at applied potentials corresponding to high adsorbed CO concentrations (Supplementary Fig. 14). We found that *U* = −1.0 V leads to the highest amount of CO adsorbed on Ni–N_2_C_2_ moiety, leading to 1985 cm^−1^ with vertical polarization. For Ni–N_3_C_1_, the highest CO concentration is for *U* = −1.1 V, leading to 1959 cm^−1^ while for Ni–N_4_, the highest CO concentration is for *U* = −1.25 V leading to 1942 cm^−1^. Both latter cases lead to CO polarization off from vertical. Our calculated values are in the range of experiment and the dependence on site is sufficiently large to identify which Ni sites are present. Additionally, we used QM calculation to predict XPS BE shifts for N 1*s* to identify how many nitrogens are bonded to Ni in Ni–SACs. The experimentally deconvoluted N 1*s* peaks indicate several nitrogen moieties, but only pyridinic nitrogen bonded to Ni shows a shift from normal pyridinic N BE value. The experimental BE difference between pyridinic N and M-N*x* varies from 0.9 to 1.3 eV.

So, we took pyridinic N 1*s* as reference BE for comparing the BE shifts. The BE shift, ΔBE for N 1*s* is 0.96 eV (lowest) for Ni–N_2_C_2_ site and 1.18 eV (highest) for Ni–N_4_ site, which are in the range of experiment (Table 2). We found that the C 1*s* BE decreases to 0.62 eV and 1.21 eV for Ni–N_3_C_1_ and Ni–N_2_C_2_. Finally, we also observed that the Ni 2*p* BE shift is 0.28 eV for Ni–N_2_C_2_ (lowest) and 0.90 eV for Ni–N_4_ site (highest). These differences might be too small to be resolved experimentally.

**Table 2:** QM derived binding energy shift for N 1*s* for different bonding sites with nickel.

Type of Ni-sites	Reference value	Ni–N_2_C_2_	Ni–N_3_C_1_	Ni–N_4_	Graphitic N
Calculated ΔBE, eV for N 1*s*	0 (pyridinic N)	+0.96	+1.05	+1.18	+2.76
Experimental^16,20,26,51^	398.0–398.5 eV	+0.9–1.3	+2.5–3.5		
Calculated BE shift for C 1*s*	0 (graphene)	−1.21	−0.62	–	–
Calculated BE shifts for Ni 2*p*	0 (Ni atom)	+0.28	+0.50	+0.90	–

## Discussion

The CO_2_ reduction to CO involves four steps. Firstly, linear CO_2_ binds to the catalysts surface (*) by forming bent *CO_2_^−^ via electron transfer

9 $$\ast + {\mathrm{CO}}_2 + {\mathrm{e}}^ - \to \ast {\mathrm{CO}}_2^ -$$

then hydrogen transfer from water converts bent CO_2_ into adsorbed *COOH intermediate plus solvated OH^−^

10 $$\ast {\mathrm{CO}}_2^ - + {\mathrm{H}}_2{\mathrm{O}} \to \ast {\mathrm{COOH}} + {\mathrm{OH}}^ -$$

then a second hydrogen transfer from water converts adsorbed *COOH intermediate into bound CO plus H_2_O plus solvated OH^−^

11 $$\ast {\mathrm{COOH}} + {\mathrm{H}}_2{\mathrm{O}} + {\mathrm{e}}^ - \to \ast {\mathrm{CO}} + {\mathrm{H}}_2{\mathrm{O}} + {\mathrm{OH}}^ -$$

Finally, the bound *CO desorbs from the catalyst surface as CO gas, and making the surface empty (*) for further reaction

12 $$\ast {\mathrm{CO}} \to \ast + {\mathrm{CO}}\,({\mathrm{g}})$$

The Tafel slope provides an indication of the rate determining step for this reaction. According to Butler–Volmer equation, Tafel slopes should be around 120, 60, 40 mV dec^−1^, and ∞ if the rate determining steps are (9), (10), (11), and (12), respectively. We calculated Tafel slopes of 52, 62, and 55 mV dec^−1^ for Ni–N_2_C_2_, Ni–N_3_C_1_ and Ni–N_4_, respectively, obtained near to their onset potential, *U*_onset._

Our calculations show that CO_2_ activation is easier due to presence of Ni^+^ instead of Ni^2+^, where the highest occupied molecular orbital (HOMO) of Ni^+^ in Ni–SACs (Ni $3d_{x^{2} - y^{2}}$ rather than $3d_{z^{2}}$ for Ni^2+^) has tendency to overlap with the C 2p* orbital in CO_2_. Thus, protonation of *${\mathrm{CO}}_2^ -$ to become *COOH leads to the highest energy barrier (Figs. 3 and 7), which is the RDS for all active sites we studied. This should lead to Tafel slope ~60 mV dec^−1^. Recently, Gu et al.^52^ observed that the initial electron for reduction of CO_2_ is decoupled from a proton transfer on Fe^3+^–N–C and found that CO_2_ adsorption is fast, and the rate limiting step is protonation of the adsorbed CO_2_^–^ to form an adsorbed COOH intermediate, with an experimental Tafel slope of 64 mV dec^−1^. Moreover, experiments by Liu et al.^29^ for CO_2_RR on a Ni-single atom catalyst confirmed the presence of Ni^+^ by operando spectroscopic characterization. They observed that the proton transfer to the adsorbed *CO_2_ is the rate determining step with a Tafel slope of 72 mV dec^−1^. Thus the experiments lead to Tafel slope very close to the 62 mV dec^−1^ we calculate for Ni–N_3_C_1_.

It has been shown experimentally^49^ that Ni-single atom catalysts can deliver a large current density of 220 mA cm^−2^ at −1.0 V RHE with 88.5% FE. We predict that our most active site at −1.0 V, Ni–N_3_C_1_, leads to a CO current of 259 mA cm^−2^, slightly higher than experiment (192 mA cm^−2^). This may be due to mass transport issues that we ignore. According to Singh et al.^53,54^ at higher negative potential (−1.0 V vs RHE), the transport of CO_2_ to the catalyst and the transport of OH^−^ anions away from the catalyst become rate limiting due to cathodic polarization. Moreover, as a consequence of the latter effect, the pH near the catalyst surface rises well above 7, and the concentration of CO_2_ decreases dramatically because of the reaction of CO_2_ with OH^−^ anions to form HCO_3_^−^ and CO_3_^−^ anions. The net effect is to alter the current-voltage profile significantly.

The double layer capacitance is an important parameter that can affect the current-voltage relations for CO_2_ reduction. The double layer capacitance arises from charge transfer in contact between the conductive electrode and the electrolytic solution. Experimentally, the double layer capacitance, *C*_diff_, is between 20 and 25 μF cm^−2^ for most metal surfaces. Ringe et al.^55^ assumed a value of 25 μF cm^−2^ in their model to address double layer effects for CO_2_ reduction reaction on Ag (111) single crystal electrode. As described earlier, the GCP-K leads directly to a differential capacitance, *C*_diff_, that can be compared to experiment. The values for various intermediates are shown in Table 3. We found that the capacity differences, *C*_diff_ are 12–29 μF cm^−2^ for Ni–N_2_C_2_, 12–20 μF cm^−2^ for Ni–N_3_C_1_, and 13–22 μF cm^−2^ for Ni–N_4_ system, close to the observed values (20–25 μF cm^−2^) reported experimentally^56,57^. This further validates our methodology for catalyst-electrolyte interface. We also calculated the PZC and *F*_0_ value for each of reaction intermediates for the various active sites (Supplementary Tables 3–5)

**Table 3:** Calculated differential capacitance for different intermediates during CO_2_ reduction reaction.

	Differential Capacitance, *C*_diff_		
Species	Ni–N_2_C_2_	Ni–N_3_C_1_	Ni–N_4_
Empty sites	15.87	20.06	17.02
CO_2_	29.00	19.85	14.88
Cis-COOH	22.66	15.24	21.93
Trans-COOH	23.79	14.64	20.30
CO	12.34	16.98	14.38
TS01	22.88	13.34	21.98
TS02	23.21	13.25	23.85
TS13	14.01	13.73	13.38
TS23	14.28	12.36	15.56
[Ni–SAC]H	15.87	13.20	19.43
[Ni–SAC]H_2_	14.91	12.70	14.30

.

In conclusion, we examined the reaction mechanism and kinetics for the reduction of CO_2_ over graphene-supported Ni-single atom catalysts (Ni–SACs) using the new grand canonical potential kinetics (GCP-K) formulation. This allows the geometry of the transition states and the charge transfer from electrode to adsorbed species to change continuously along the reaction coordinates as the potential is changed. GCP-K describes the electron transfer accompanying proton or hydrogen transfer as a continuous process, rather than as a discrete electron jump as in the PCET formulation of traditional Butler–Volmer kinetics.

We applied the GCP-K method to determine the reaction mechanism and kinetics for CO_2_RR on all three Ni sites: Ni–N_2_C_2_, Ni–N_3_C_1_, and Ni–N_4_ likely to be present in Ni–SACs. We find each site leads to unique kinetics with Ni–N_2_C_2_ dominant for −0.93 < *U* < −0.75 V, producing 10 mA cm^−2^ current density at *U*_onset_ = −0.84 V, with a Tafel slope of 52 mV dec^−1^, a faradic efficiency (FE) of 98%, and TOF = 3903 h^−1^ per Ni site. On the other hand, Ni–N_3_C_1_ dominant for −1.05 < *U* < −0.93 V producing 10 mA cm^−2^ current density at *U*_onset_ =  −0.92 V, with a Tafel slope of 62 mV dec^−1^, a FE = 78% and TOF = 2940 h^−1^ per Ni site while Ni-N_4_ dominant for *U* < −1.05 V producing 10 mA cm^−2^ current density at *U*_onset_ =  −1.03 V with a Tafel slope of 55 mV dec^−1^, a FE = 99% faradic efficiency, and TOF = 3944 h^−1^ per Ni site. These predicted overall kinetics show reasonable agreement with experimental studies. Thus ref. ^25,27^ leads to CO dependence on *U* similar to what we find for Ni–N_2_C_2_. We find that HER activity on Ni–SAC depends very much on the site, with HER on Ni–N_3_C_1_ starting at *U* = −0.8 V. The Ni–N_4_ leads to the best overall performance for CO_2_ reduction: At *U* = −1.05 V we find 40 mA cm^−2^ with CO FE ~ 100%.

In order to help guide identification of the sites in experimental synthesized Ni–SACs, we predicted the XPS N and C 1*s* and Ni 2*p* BE shift and the CO vibrational frequencies for various sites. We find that the pyridinic N 1*s* XPS binding shifts is highest (+1.18 eV) for Ni–N_4_ and lowest (+0.96 eV) for Ni–N_2_C_2_. While the C 1*s* shift is −0.62 for Ni–N_3_C_1_ and −1.21 eV for Ni–N_2_C_2_. Finally, The Ni 2*p* BE shift is +0.90 eV (maximum) for Ni–N_4_ and +0.28 eV (minimum) for Ni–N_2_C_2_ site. The CO stretch for CO bound to Ni is 1985 cm^−1^ at −1.0 V on Ni–N_2_C_2_ site and 1942 cm^−1^ at −1.25 V on Ni–N_4_ site. These predicted results for Ni–N_2_C_2_ site agree best with experiment, but we expect that most synthesized catalysts have mixtures of all three Ni sites so that the total CO current for arises from each of them.

## Methods

### Computational details

The geometry optimization was performed as a function of constant charge using the PBE-D3 functional^58–60^ with VASPsol^61,62^ implemented in VASP^63–65^ and including spin polarization (Supplementary Note 2). Then we used the Legendre transformation to transform the VASP results to constant potential followed by using the CANDLE solvation model^35^ (as implemented in jDFTx v.1.2.1)^66^ for the final constant potential results. Here we included a few waters to describe explicitly the polarization involved in proton transfer. The positions of these waters were optimized with VASPsol. For the reaction barriers we did free energy calculation for each point along the NEB reaction path using VASPsol, so that the geometry of the transition state could change adiabatically as a function of applied potential. The VTST package^67^ with climbing image nudged elastic band (CI-NEB)^68^ was used to obtain the reaction pathway and transition states including solvation.

We used a plane wave basis set with 500 eV cutoff energy. The geometry optimization criteria were set to 10^−6^ eV for energy and 0.01 eV Å^−1^ for forces on each atom while for CI-NEB we used 0.02 eV Å^−1^. A Gaussian smearing of 0.05 eV was applied throughout the whole calculation. The K-points were selected to be 5 × 5 × 1 for structure relaxation and energy calculations. The graphene unit cell dimensions were optimized to be 2.46 Å × 2.46 Å with 20 Å distance between layers perpendicular to the surface. Finally, 4 × 4 super cells of graphene were used with 26 carbon atoms and 1 metal atom placed on the carbon divacancy.

The single point jDFTx calculations were used to obtain the combined DFT and solvation free energy using the geometry optimized with VASPsol. The jDFTx calculation was chosen because of the accuracy in describing continuum solvation and for including constant potentials for electrochemical reaction. But we found that a single point calculation is sufficient for the stable species since the structures observed are similar in both VASPsol and jDFTx (Supplementary Table 6). The CANDLE solvation model was used in jDFTx to describe solvation implicitly. Using this scheme, $\mu _{e,{\mathrm{SHE}}}$ = 4.66 eV, which was used for all structures. A plane wave basis set with energy cutoff of 20 Hartree was used with a k-point mesh of 5 × 5 × 1. The free energy convergence criteria were set to 10^−8^ Hartree.

XPS BE shift calculations were performed using the core level energies implemented in VASP^63^. There are two methods for such calculations; we used simpler option (ICORELEVEL = 1) to calculate the core levels in the initial state approximation. This is based on the observation in recent publications that this method leads relative BE shifts in good agreement with experimental XPS^69,70^. This method involves recalculating the KS eigenvalues of the core states after a self-consistent calculation of the valence charge density. The phonon density of states were calculated using VASP by setting IBRION = 5 in the INCAR file. The Hessian matrix and the vibrational frequencies of a system were calculated using atom displacements in all three cartesian directions with a step size of 0.015 Å (POTIM). We allowed only the adsorbed molecule to vibrate and the vibrational frequencies were taken from the normal mode analysis. To calculate entropy, we used the phonon density of states, as found in OUTCAR as “*f*” on the real axis. Then we extracted total vibrational ZPE, enthalpy, and entropy.

## Supplementary information

Supplementary Information

Description of Additional Supplementary Files

Supplementary Data 1

## Data Availability

The data that support the findings of this study are available from the corresponding author upon request.
