# Supplementary material for: Reaction mechanism and kinetics for CO2 reduction on nickel single atom catalysts from quantum mechanics
Source: Nat Commun. 2020 May 7;11:2256. doi: 10.1038/s41467-020-16119-6 (PMC7205999; doi:10.1038/s41467-020-16119-6)
Supplement: Supplementary file 1 — Supplementary Information [file 41467_2020_16119_MOESM1_ESM.pdf]

Supplementary Information

Reaction Mechanism and Kinetics for CO_2 Reduction on Nickel Single Atom Catalysts from Quantum Mechanics

Hossain et al.

Supplementary Note 1

Derivation of GCP (U) equation

In the GCP-K formulation, the free energy *F(n)* and GCP (U) can be obtained from either constant charge or constant potential calculations. In this study, we first calculated *F(n)* by QM method at different charges, and then fitted the quadratic curve by using the equation of $F(n) = a(n - n_0)^2 + b(n - n_0) + c$, where a, b, and c are fitted parameters. After fitting we obtain the physical parameters, as $c = F_0$, $b = \mu_{e,SHE} - eU_{PZC}$, and $a = -\frac{1}{2C_{diff}}$.

$$F(n) = a\,(n - n_0)^2 + \; b\,(n - n_0) + c \qquad (1)$$

$$G\,(n, U) = F\,(n) - ne\,(U_{SHE} - U) \qquad (2)$$

Using equation (1)

$$G\,(n, U) = a\,(n - n_0)^2 + \; b\,(n - n_0) + c - \; ne\,(U_{SHE} - U) \qquad (3)$$

As we know from the minimization, $\frac{dG\,(n;U)}{dn} = 0$

» $2a\,(n - n_0) + b - e\,(U_{SHE} - U) = 0$

» $(n - n_0) = \frac{e\,(U_{SHE} - U) - b}{2a}$

» $n = \; n_0 + \frac{e\,(U_{SHE} - U) - b}{2a}$

From (3) we get,

$$G\,(n, U) = a\,\{\frac{e\,(U_{SHE} - U) - b}{2a}\}^2 + b\;\frac{e\,(U_{SHE} - U) - b}{2a} + c + \{n_0 + \;\frac{e\,(U_{SHE} - U) - b}{2a}\}\;e\,(U_{SHE} - U) \quad (4)$$

$$GCP\,(U) = -\frac{1}{4a}(b - \mu_{e,SHE} + eU)^2 + \; c - n_0\mu_{e,SHE} + n_0 eU \quad [eU_{SHE} = \; \mu_{e,SHE}] \qquad (5)$$

Supplementary Note 2

Spin effect evaluation

To study the possibility of spin polarization for hybrid DFT, we simplified the system to the finite Ni doped graphene-like system below and carried out B3LYP-D3 hybrid functional calculations using Jaguar. This used the Los Alamos core-valence effective core potential (describing Ni with 18 explicit electrons) with the LACV3P**++ basis set. For the Ni-N_4 system without ligand we found that the ground state is a singlet with no spin polarization. The lowest triplet state is higher by 0.95 eV; it has *pz* singly occupied orbitals. This suggests that the configuration on the Ni is closed shell *d*8 with doubly occupied *xy*, *yz*, *xz,* and z^2 *d*-orbitals and empty x^2-y^2 overlapping the 4 N sp^2 lone pairs pointing at the Ni. Here the triplet corresponds to a graphene π to π^* transition. We also carried out the PBE-D3 calculation with Jaguar and found a ground state singlet with the triplet 0.91 eV higher.

In the VASP PBE-D3 calculations, the ground state was closed shell even though we allowed spin polarization. To explore further the possibility of open shell character, we required an Ms=1 state (triplet) and found an energy 0.25 eV higher with the unpaired spins on the graphene. Thus we conclude that the ground state is correctly described in VASP PBE-D3.

For the most important intermediate (cis-COOH), B3LYP-D3 produces a doublet ground state with the unpaired spin in the x^2-y^2 orbital. The lowest quartet state is 0.28 eV higher with unpaired spins also in the π and π^* orbitals. We interpret this as the radical C of (HO)C=O forming a covalent bond to the Ni triplet excited state that starts with a hole in the d_z^2 and an electron in the x^2-y^2. This leads to a covalent C-Ni σ bond leaving the unpaired spin in the x^2-y^2 orbital. For PBE-D3 we found the doublet ground state with the quartet 0.67 eV higher. Thus for the Ni-N_4 system with and without ligand, PBE-D3 and B3LYP-D3 predict the same trend.

In the VASP PBE-D3 calculations on the (cis-COOH) system, we found a closed shell description, with partial occupation of the x^2-y^2 orbital. Thus we conclude that the PBE-D3 description is adequate.

Supplementary Flow Chart 1

The procedure shows how to obtain the grand canonical potential as a function of fixed potential.

Geometry optimization using VASPsol as a function of charge $(n - n_0) = n_1, n_2, n_3, \dots$

↓

Single point calculation using same charge in jDFTx with CANDLE solvation method to get the Free energies (F_1, F_2, F_3 ……) as a function of charge $(n_1, n_2, n_3, \dots)$ respectively

↓

Finally, GCP (U) obtain by the minimization of grand canonical free energies, G (n; U) via Legendre transformation which converts fixed charge free energy, *F (n)*, to fixed potential grand canonical potential, GCP(U) according to following equation

$$\begin{aligned} GCP\ (U) &= \min G\ (n; U) \\ &= \min\ \{F(n) - ne\ (U_{SHE} - U)\} \end{aligned}$$

Supplementary Table 1. Calculation procedure of GCP (U) value based on fixed potential.

Quadratic fitting of G (n, U) as a function of charge (n_1, n_2, n_3 …), $ax^2 + bx + c = y$			Number of electrons at neutral, n_0	Electron at minimization of G_{min}, $n_{min} = (n-n_0) = -(b - U) / 2a$	pH = H<br>-U = **U_1** vs RHE	U = (U_1+ H*0.0591)	ZPE + Hvib - TSvib	GCP (U)
a	b	c			G (n, U) = $a*n_{min}^2 + (b-U)*n_{min} + c$			
a_1	b_1	c_1	n_{01}	n_{min1}	G_1 (n, U)		V_1	G_1 (n, U) + V_1
a_2	b_2	c_2	n_{02}	n_{min2}	G_2 (n, U)		V_2	G_2 (n, U) + V_1

Here we show a simple demonstration of our new grand canonical potential calculation by applying constant potential method. We initially obtain the free energy as a function of constant charge method, then we minimize the free energy quadratic equation as a function of applied potential (U_1 vs RHE). Then, we obtain the direct dependence of applied potential (U_1 vs RHE) on GCP (U) value. As the applied potential (U_1) is changed, it leads to changes in the charges within the system as in the relation of U and n_{min}.

Supplementary Table 2. The relation between applied potential and corresponding charges within the system observed for different reaction intermediates/TS during CO_2 at pH 7 and Ni-N_4 system.

Species	#of elec. at G_{min} for 0 V vs RHE	#of elec. at G_{min} for 1 V vs RHE
(CO_2+H_2O) Reactant, 0	-1.0*	0.70
Cis-COOH Product, 1	0.62	1.70
trans-COOH Product, 2	0.78	1.80
TS01	0.58	1.66
TS02	0.75	1.77
(Cis-COOH +H_2O)	-0.80	0.80
(trans-COOH +H_2O)	-0.50	0.90
CO product, 3	0.70	1.48
TS 13	0.42	1.20
TS23	0.76	1.54

*- sign refers less electron than neutral system and TS = transition state

Supplementary Table 3. Parameters obtained from quadratic fitting to obtain the Grand Canonical Potential or Free Energy for Ni-N_4 system.

Species	$a = \frac{1}{2C_{diff}}$ (eV/electron^2)	Differential Capacitance, C_{diff}(μF/cm^2) calculated from "a" parameter	$b = (\mu_{e,SHE} - eU_{PZC})$ (eV/electron)	c = F_0 (eV)	Vibrational contrib. (ZPE + Hvib - TSvib), kcal/mol
CO_2	6.41E-01	14.88	-3.68E+02	2.21E+04	50.00
Cis-COOH	4.35E-01	21.93	-1.58E+02	2.99E+03	47.70
Trans-COOH	4.70E-01	20.30	-1.71E+02	4.20E+03	46.35
CO	6.63E-01	14.38	-3.54E+02	2.11E+04	52.28
TS01	4.34E-01	21.98	-1.58E+02	2.95E+03	47.71
TS02	4.00E-01	23.85	-1.46E+02	1.86E+03	46.35
TS13	7.13E-01	13.38	-2.61E+02	1.25E+04	50.65
TS23	6.13E-01	15.56	-2.25E+02	9.23E+03	53.65
[Ni-SAC]H	4.91E-01	19.43	-1.39E+02	7.62E+02	48.96
[Ni-SAC]H_2	6.67E-01	14.30	-2.12E+02	6.36E+03	45.09

$\mu_{e,SHE}$ is the chemical potential of an electron vs. SHE, U_{PZC} is the potential of zero net charge, and F_0 is the free energy at zero net charge. Quote the U_{PZC} explicitly rather than b

Supplementary Table 4. Parameters obtained from quadratic fitting to obtain the Grand Canonical Potential or Free Energy for Ni-N_3C_1 system.

Species	$a = \frac{1}{2C_{diff}}$ (eV/electron^2)	Differential Capacitance, C_{diff}(μF/cm^2) calculated from "a" parameter	$b = (\mu_{e,SHE} - eU_{PZC})$ (eV/electron)	c = F_0 (eV)	Vibrational contrib. (ZPE + Hvib - TSvib), kcal/mol
CO_2	4.81E-01	19.85	-7.55E+02	5.40E+04	50.00
Cis-COOH	6.26E-01	15.24	-2.49E+02	1.03E+04	47.700
Trans-COOH	6.52E-01	14.64	-2.65E+02	1.17E+04	46.35
CO	5.62E-01	16.98	-1.18E+02	-1.08E+03	52.28
TS01	7.15E-01	13.34	-2.48E+02	1.02E+04	47.70
TS02	7.20E-01	13.25	-2.50E+02	1.04E+04	46.35
TS13	6.95E-01	13.73	-5.83E+02	3.95E+04	50.65
TS23	7.72E-01	12.36	-4.07E+02	2.42E+04	53.60
[Ni-SAC]H	7.23E-01	13.20	-5.28E+02	2.85E+04	48.96
[Ni-SAC]H_2	7.51E-01	12.70	-2.92E+02	1.27E+04	45.09

Supplementary Table 5. Parameters obtained from quadratic fitting to obtain the Grand Canonical Potential or Free Energy for Ni-N_3C_1 system.

Species	$a = \frac{1}{2C_{diff}}$ (eV/electron^2)	Differential Capacitance, C_{diff}(μF/cm^2) calculated from "a" parameter	$b = (\mu_{e,SHE} - eU_{PZC})$ (eV/electron)	c = F_0 (eV)	Vibrational contrib. (ZPE + Hvib - TSvib), kcal/mol
CO_2	3.29E-01	29.00	-1.13E+02	-1.53E+03	50.00
Cis-COOH	4.21E-01	22.66	-1.45E+02	1.35E+03	47.700
Trans-COOH	4.01E-01	23.79	-1.45E+02	1.35E+03	46.35
CO	7.73E-01	12.34	-2.69E+02	1.21E+04	52.28
TS01	4.17E-01	22.88	-1.45E+02	1.35E+03	47.70
TS02	4.11E-01	23.21	-1.46E+02	1.35E+03	46.35
TS13	6.81E-01	14.01	-2.37E+02	9.32E+03	50.65
TS23	6.68E-01	14.28	-2.17E+02	9.42E+03	53.60
[Ni-SAC]H	6.01E-01	15.87	-1.68E+02	2.85E+03	48.96
[Ni-SAC]H_2	6.40E-01	14.91	-2.03E+02	5.73E+03	45.09

$$\text{Differential capacity, } C_{diff} = \frac{1.6\times10^{-19}\times10^{6}}{2a\times8.385\times10^{-15}}\ \mu F/cm^2$$

Where, "a" is the parameter obtained from quadratic equation and area of 4×4 graphene cell is 8.385 x 10^{-15} cm^2.

Supplementary Table 6. Comparison between the two different codes used for geometry optimization.

Optimized molecules	VASP + VASPsol	jDFTx + CANDLE
d(O-H) in H_2O	0.9731 Å	0.9839 Å
θ(HOH) in H_2O	104.62°	106.87 Å
d(O-C) in CO_2	1.1699 Å	1.1716 Å
d(O-C) in CO	1.1424 Å	1.1387 Å

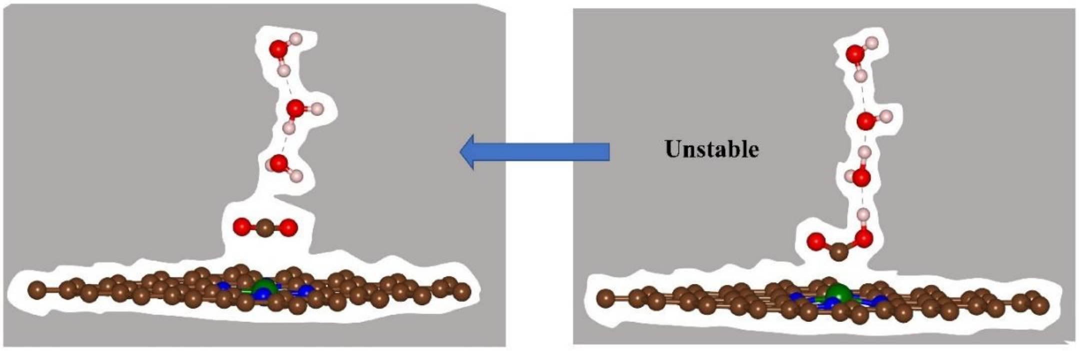

Supplementary Figure 1. Proposed initial models for CO_2 reduction reaction (CO_2 to COOH reaction). We found that the cis-COOH (right figure) structure is not stable during geometry optimization in VASPsol instead of it converts into CO_2 (left).

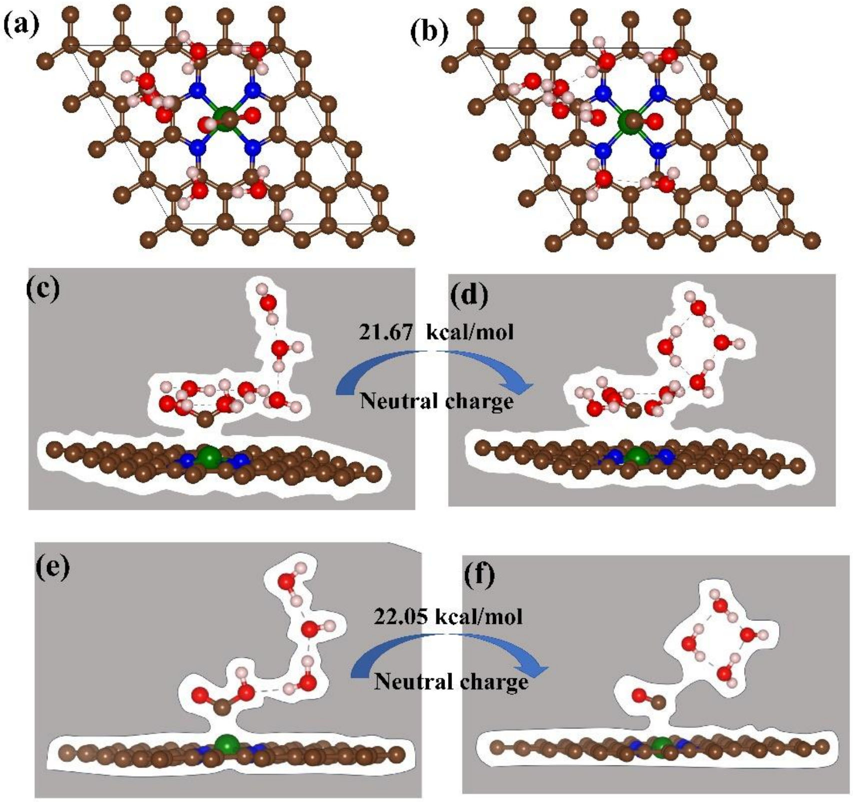

Supplementary Figure 2. Comparison of the predicted energy barrier for two explicit water models. (a, b) Top and (c, d) side view of COOH and CO models including more explicit waters and (e, f) side view of our used models for the system of COOH to CO conversion. We compare the energy barrier for the system with more explicit water (six) molecules with our model (three water molecules). We found that both systems have same energy barrier, but more waters make the system computationally expensive.

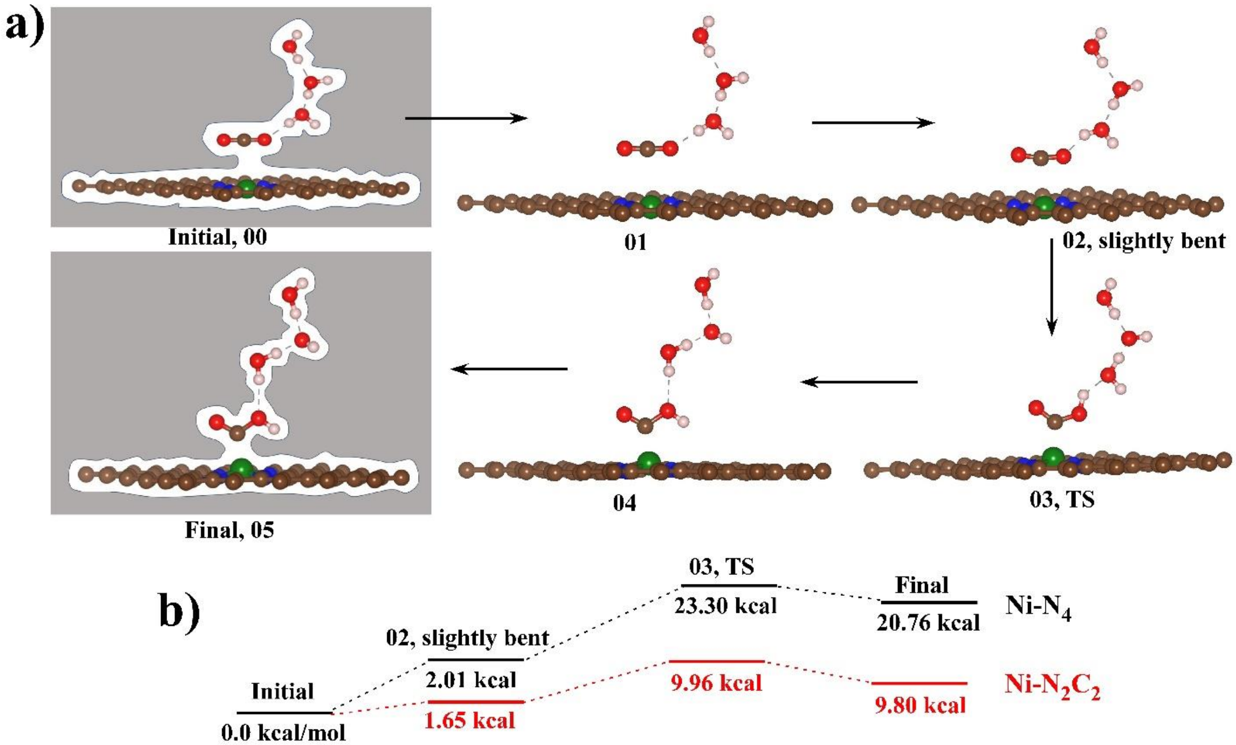

Supplementary Figure 3. Reaction pathways for linear CO_2 to trans-COOH intermediate at U= -0.8 V applied potential. (a) The minimum energy path calculation using the Climbing Image NEB (CINEB) method with implicit solvation, and (b) Reaction energetics and TS barrier for the protonation step for Ni-N_4 and Ni-N_2C_2 sites at -0.8 V vs RHE applied potential. This reaction path involves lower energy barrier than cis-COOH case. For Ni-N_2C_2, the linear CO_2 first becomes slightly bent at the 02 image leading to a low energy barrier (1.65 kcal/mol for Ni-C_2N_2 at U= -0.8V), indicating fast decoupled electron transfer followed by proton transfer with higher energy barrier (8.31 kcal) at image 03. Similarly, For Ni-N_3C_1 and Ni-N_4, we find 1.87 and 2.01 kcal/mol energy barrier to form slightly bent CO_2 and then overcome the proton transfer barrier of 12.75 and 21.29 kcal to form OCOH respectively at -0.8 V potential.

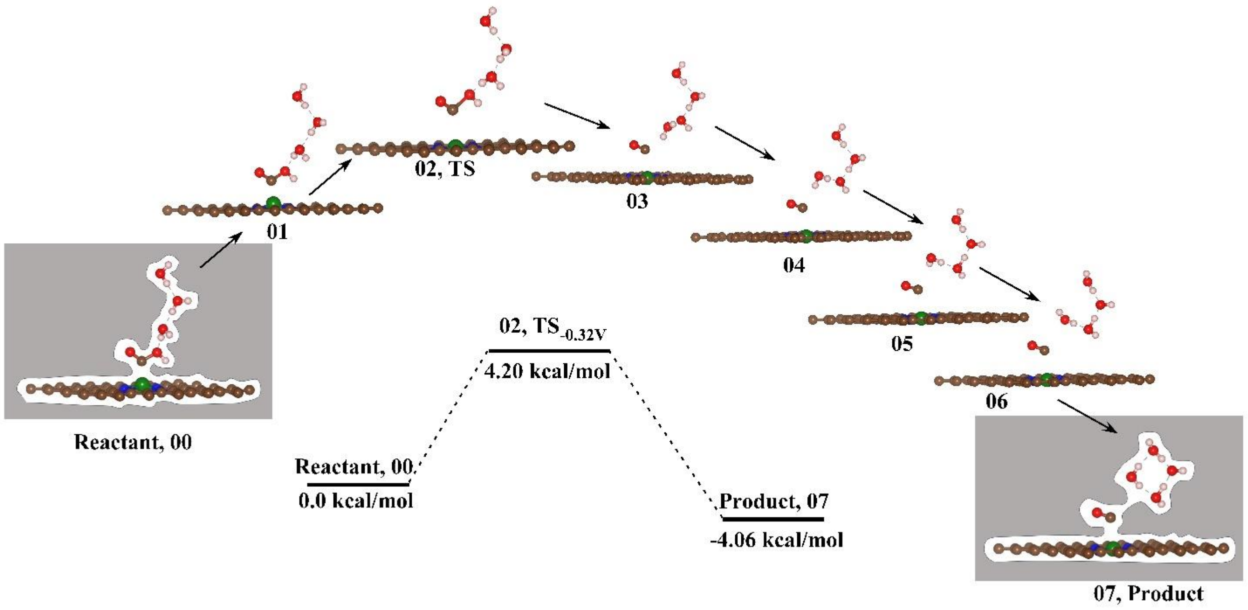

Supplementary Figure 4. Climbing image NEB path for the conversion of trans-COOH to CO product at -0.32 V applied potential on Ni-N_4 sites. The conversion of trans-COOH to CO involves 4.2 kcal/mol reaction barrier due to the breakage of OC-OH bond in COOH at -0.32 V vs RHE at neutral conditions.

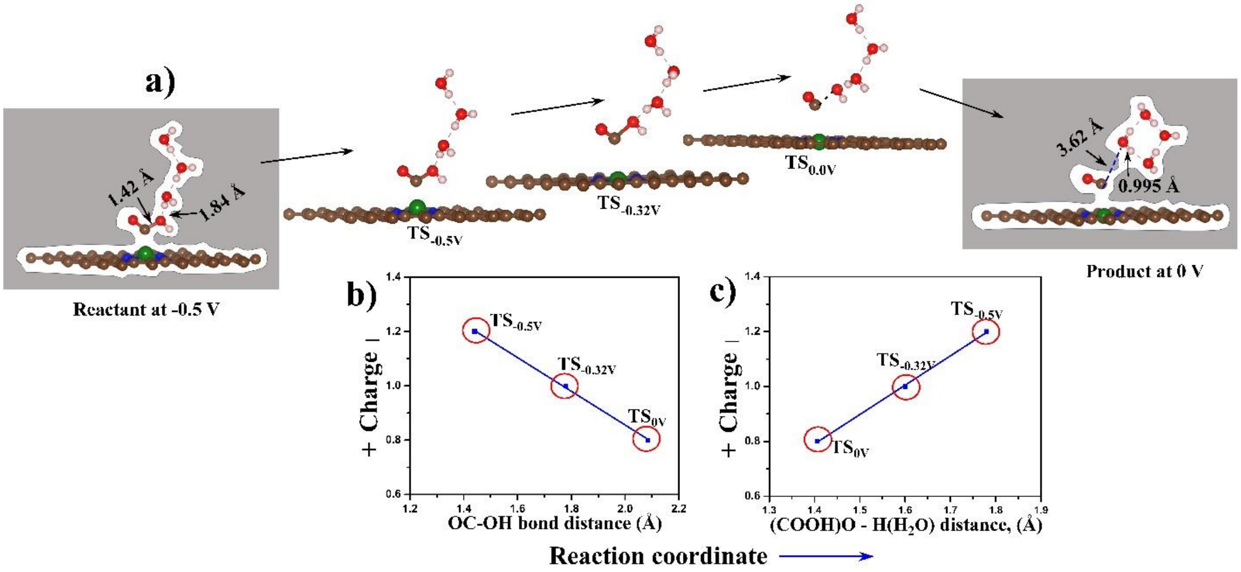

Supplementary Figure 5. Transition states (TS) change as a function of applied potential for the trans-COOH to CO formation step. (a) TS moving toward the reactant with decreasing reaction barrier as a function of potential, (b) and (c) Reaction coordinates changes linearly with charges on the TS as the potential is applied to initiate the reduction process. The transition state at zero potential is close to the product (OC-OH_2 is 3.62 Å) while with applied potential it moves towards the reactant. The initial bond distance at 0 V (2.19 Å) between OC-OH in the trans-COOH TS decreases linearly with applied potential, reaching 1.44 Å at -0.5 V (b). In contrast the distance between O(COOH)-H(H_2O) gradually increases with potential (c). Compared to the cis-COOH to CO path, the trans-COOH path has a lower energy barrier, requiring less overpotential to overcome the barrier because of the extra charge initially in the trans-COOH system. The charges within the TS species vary linearly with potential as reaction progresses in the forward direction.

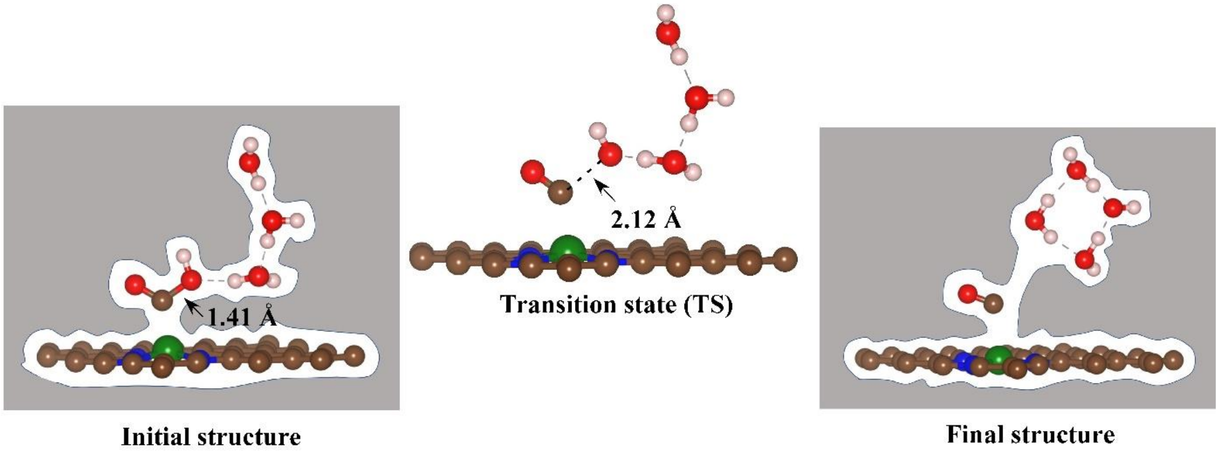

Supplementary Figure 6. The conversion of cis-COOH to CO at -0.80 V applied potential on Ni-N_3C_1. The transition state has 2.12 Å OC-OH bond distance and 6.65 kcal/mol reaction barrier.

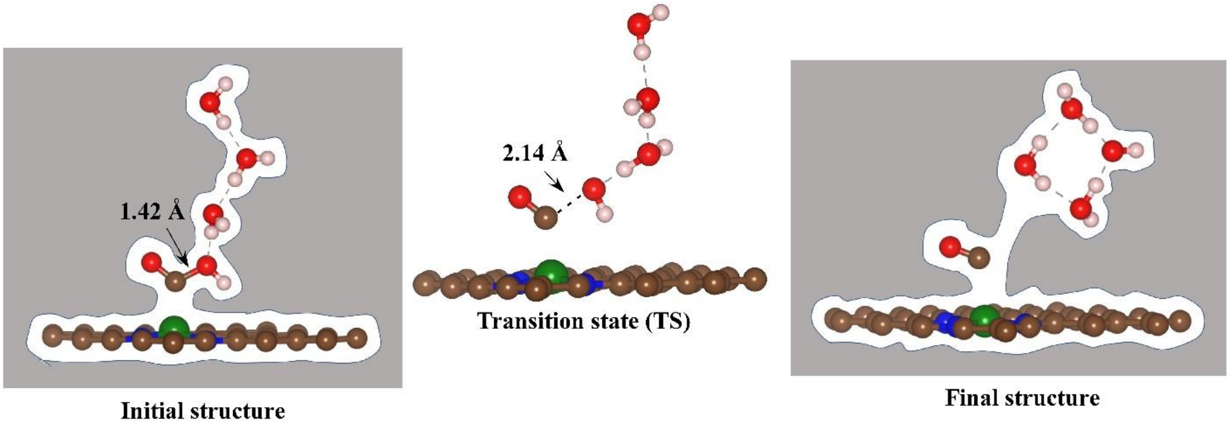

Supplementary Figure 7. The conversion of trans-COOH to CO at -0.8 V applied potential on Ni-N_3C_1. The transition state has 2.14 Å OC-OH bond distance and 7.88 kcal/mol reaction barrier.

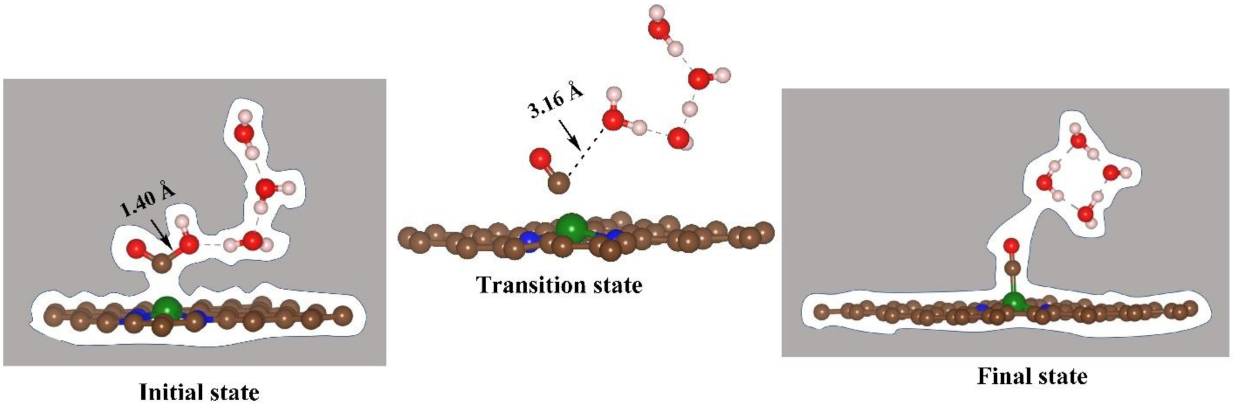

Supplementary Figure 8. The conversion of cis-COOH to CO at -0.8 V applied potential on Ni-N_2C_2. The transition state has 3.16 Å (intermediate) OC-OH bond distance and 6.29 kcal/mol reaction barrier.

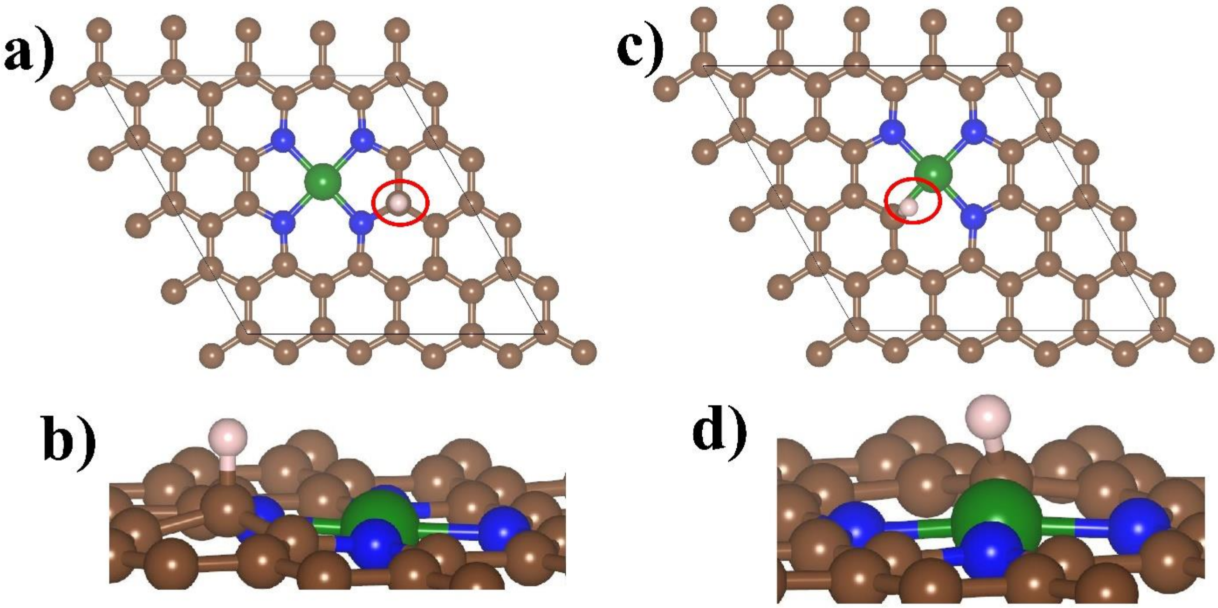

Supplementary Figure 9. Favorable sites for hydrogen evolution reaction on Ni-N_4 and Ni-N_3C_1 sites at 298K and pH 7. On Ni-N_4 - (a) Carbon next to nitrogen is most active for hydrogen adsorption, (b) same at higher resolution, and on Ni-N_3C_1 - (c) The bridge between Ni-carbon shows most favorable sites for hydrogen adsorption, (d) same at higher resolution.

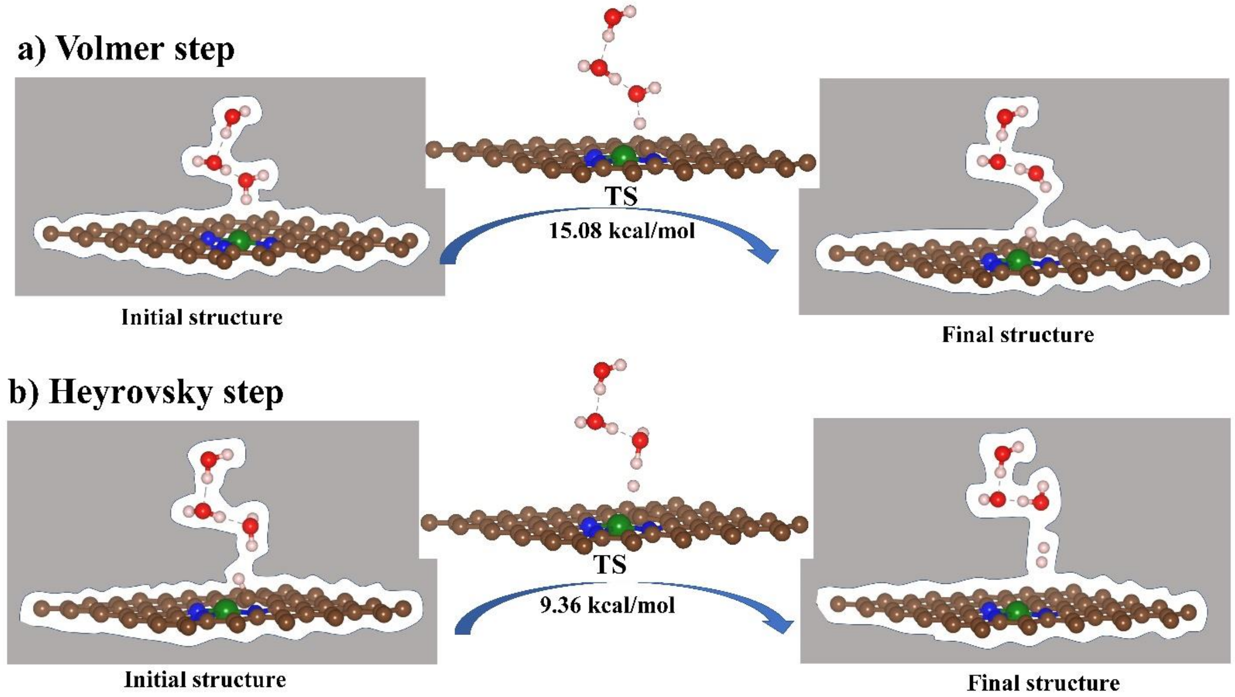

Supplementary Figure 10. Hydrogen evolution process on active bridge sites of Ni-N_3C_1 system at -0.8 V potential and neutral electrolytic condition. (a) Hydrogen adsorption step or Volmer step has a barrier of 15.08 kcal/mol while (b) Heyrovsky reaction or desorption step has 9.36 kcal/mol energy barrier at -0.8 V potential vs RHE conditions.

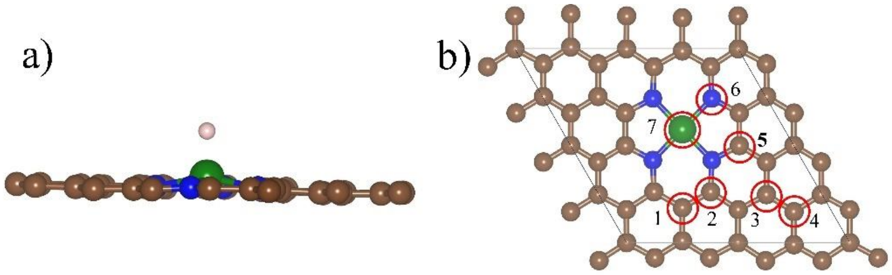

c) **Hydrogen binding energies on different sites of Ni-SAC** ($B.E._{H} = Energy_{(Ni\text{-}SAC\text{-}H)} - E_{(Ni\text{-}SAC)} - E_{(1/2H_2(g))}$)

Binding energy at site 1, $B.E._{1}$ = 23.57 **kcal/mol**

$B.E._{2}$= 23.85 **kcal/mol**

$B.E._{3}$= 23.11 **kcal/mol**

$B.E._{4}$= 28.25 **kcal/mol**

$B.E._{5}$= 20.29 **kcal/mol**

$B.E._{6}$= 20.70 **kcal/mol**

$B.E._{7}$= 30.63 **kcal/mol**

Supplementary Figure 11. Different actives sites for hydrogen adsorption on Ni-N_4 system. (a) The hydrogen atom adsorbed on Ni sites of Ni-N_4 and (b) Different adsorption sites on Ni-SAC for hydrogen and (c) The comparison of different binding energies on different sites of Ni-SAC.

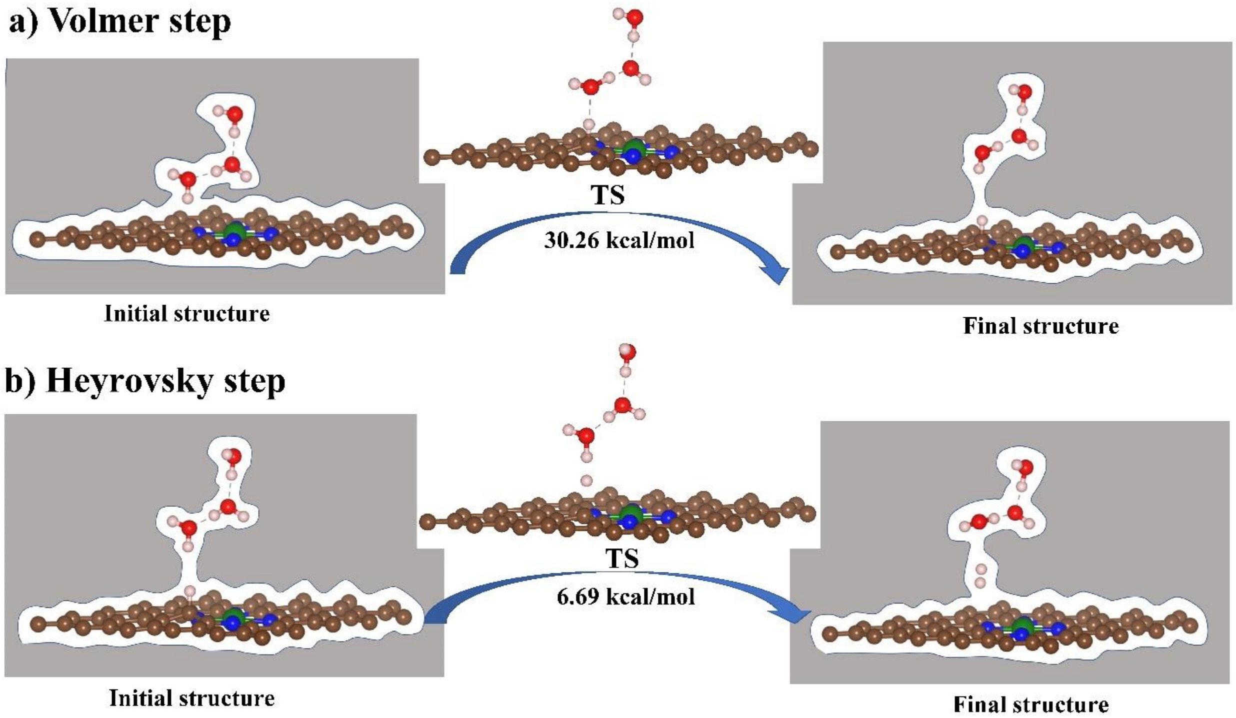

Supplementary Figure 12. Hydrogen evolution reaction occurs on active carbon sites of Ni-N_4 system at -0.8 V potential and neutral electrolytic condition. (a) Volmer step shows high absorption barrier of 30.26 kcal/mol followed by (b) Heyrovsky reaction as a desorption step with the barrier of 6.69 kcal/mol at -0.8 V vs RHE conditions.

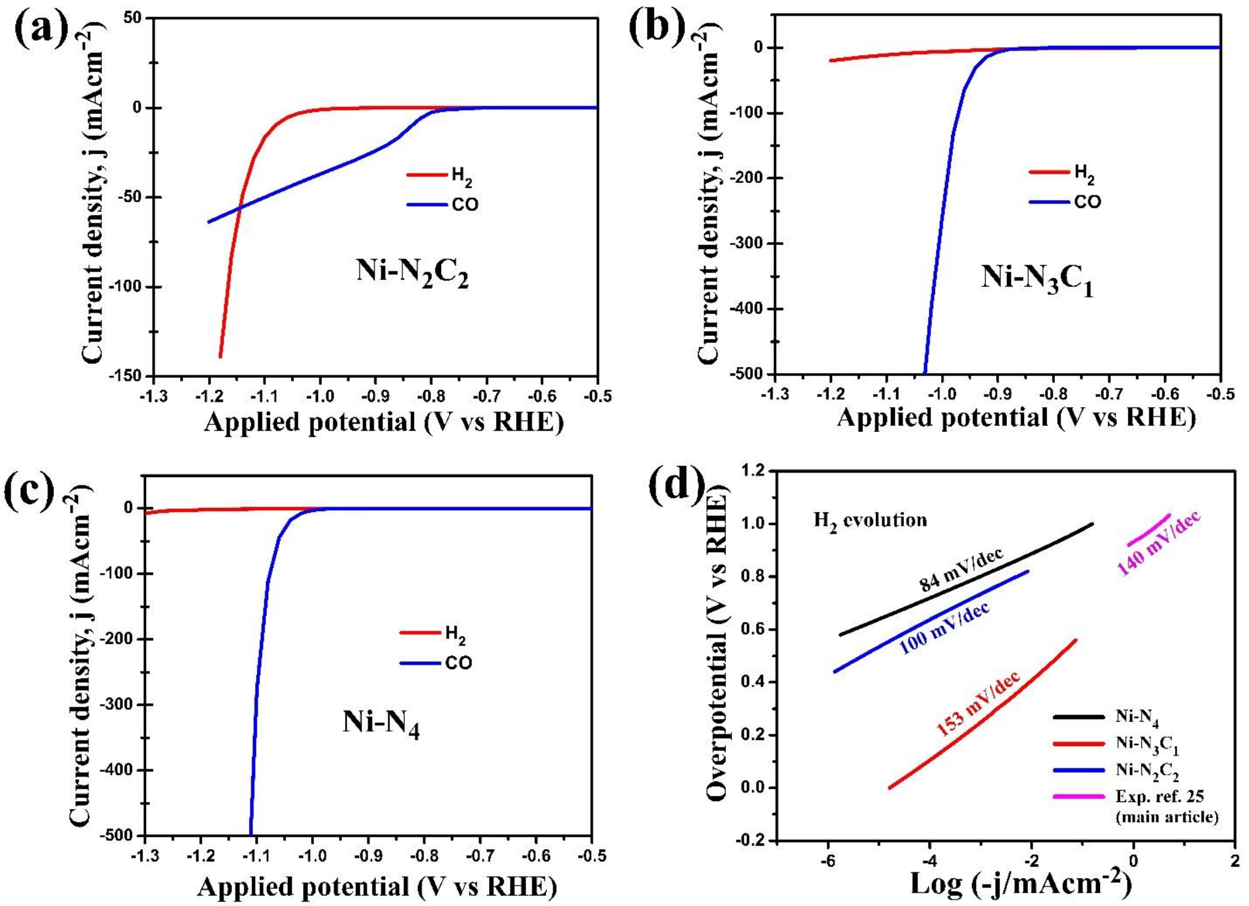

Supplementary Figure 13. QM derived current density vs applied potential for CO and H_2 evolution reaction and their Tafel slopes. Comparison of current densities between CO and H_2 evolution for (a) Ni-N_2C_2 sites, (b) Ni-N_3C_1 sites and (c) Ni-N_4 sites. The faradic efficiency increases to 100% after -1.0 V potential for Ni-N_3C_1 and Ni-N_4 case while it decreases to 0 % after -1.08 V potential for Ni-N_2C_2 sites. (d) Our derived Tafel slopes for hydrogen evolution reaction on different Ni sites obtained from I-V curves are comparable with experimental data.

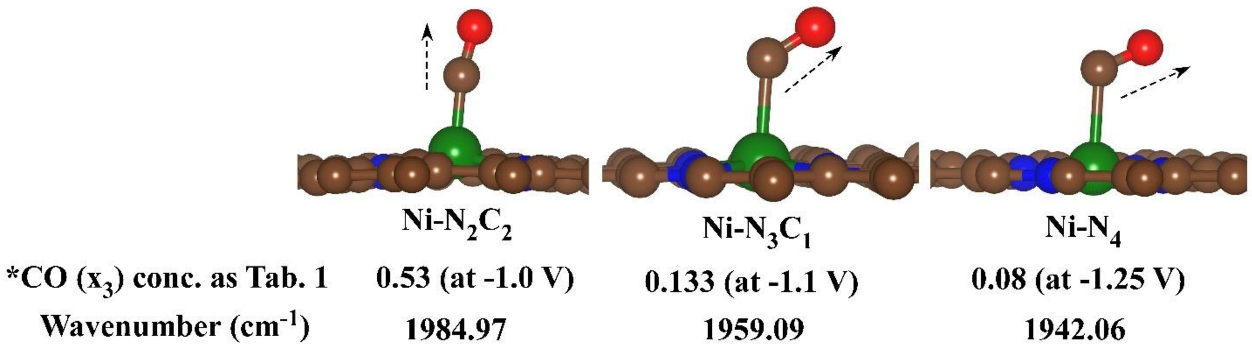

Supplementary Figure 14. QM derived CO stretch mode on different Ni sites. The absorbed CO on Ni-N_2C_2 moieties vibrates with 1985 cm^{-1} along vertical polarization at -1.0 V applied potential. For Ni-N_3C_1 CO concentration is 0.133 at U= -1.1 V, leading to 1959 cm^{-1} while for Ni-N_4 the highest CO concentration is for U= -1.25 V leading to 1942 cm^{-1}. Both latter cases lead to CO polarization off from vertical.

Supplementary Note 3

Current density Calculation

Density of carbon in graphene unit cell, $\rho_C = \frac{2}{Area}$ = 3.82 × 10^{15} cm^{-2}

Density of Ni in our model (3.22%), $p_{Ni} =$ 1.23 × 10^{14} cm^{-2}

Current density calculation, j (mA/cm^{-2}) = $n\ F\ \chi_{CO}\kappa_{CO}\rho_{Ni} \times 1000$

Where, n, number of electrons; F= Faradays constants, 96485.3/6.023×10^{23}; χ_{CO}, Concentration of CO species at constant potential; κ_{CO}, Rate constants for CO formation equation at constant potential; ρ_{Ni}, Nickel active sites density from calculation.

Current density normalization according to experiment findings,

With 1.53% Ni conc., and 0.2 mg catalysts loading contains (experimental) = 3.14 × 10^{16} active Ni sites/cm^2.

So, normalized current density for 3.14 × 10^{16} active sites is, j (mA/cm^2) =

$$\frac{\text{Current obtained from theoritical calculation } \times \text{ Total number of Ni active sites (exp. calc. value)}}{\text{Total number of Ni active sites obtained theoriticallly}}$$

Turn over frequency (TOF) Calculation

Catalyst loading: 0.2 mg/cm^2 where the Ni content was 1.53 wt%

Ni-content: 0.00306 mg/cm^2

Ni active sites density, ρNi = 0.00306 (mg/cm^2) × N_A/58693.4 (mg)

Total number of CO formation, TON = $\frac{J\ (A) \times t\ (s) \times FE_{CO}}{2F}$ cm^{-2} h^{-1}

Turn over frequency, TOF = $\frac{TON}{\rho_{Ni}}$ h^{-1}
