# Supplementary material for: Reaction mechanism and kinetics for CO2 reduction on nickel single atom catalysts from quantum mechanics
Source: Nat Commun. 2020 May 7;11:2256. doi: 10.1038/s41467-020-16119-6 (PMC7205999; doi:10.1038/s41467-020-16119-6)
Supplement: Supplementary file 2 — Description of Additional Supplementary Files [file 41467_2020_16119_MOESM2_ESM.docx]

**File Name**: Supplementary Data 1

**Description**: Supplementary Data 1 consists of optimized geometries (VASP_POSCAR format) of the intermediates and transition states (TS) for CO_2_ reduction reaction on different Ni single sites (Ni-N_4_, Ni-N_3_C_1_, Ni-N_2_C_2_) at different applied charges. (Positive number indicates excess charge than neutral system)
