# Supplementary material for: Reaction mechanism and kinetics for CO2 reduction on nickel single atom catalysts from quantum mechanics
Source: Nat Commun. 2020 May 7;11:2256. doi: 10.1038/s41467-020-16119-6 (PMC7205999; doi:10.1038/s41467-020-16119-6)
Supplement: Supplementary file 3 — Supplementary Data 1 [file 41467_2020_16119_MOESM3_ESM.pdf]

Supplementary Data 1

Optimized Geometries (VASP_POSCAR format)

Reaction Mechanism and Kinetics for CO_2 Reduction on Nickel Single Atom Catalysts from Quantum Mechanics

Hossain et al.

Ni-N_4_CO_2 (0.5 charge)

1.00000000000000

9.8400001526000000   0.0000000000000000   0.0000000000000000

-4.9200000763000000   8.5216901053999994   0.0000000000000000

0.0000000000000000   0.0000000000000000   20.0000000000000000

C   N   O   Ni   H

27   4   5   1   6

Direct

0.0064163769935362 0.0071586197981824 0.1035922068957283

0.0902116462969320 0.1761257272423610 0.1036392214636662

0.2605586743581300 0.0161480254078426 0.1037177304508602

0.3458861879768601 0.1868282112463150 0.1033267738415773

0.5058114532697332 0.0072556078699804 0.1034159003009788

0.5908937463053230 0.1762051142527649 0.1033994477186325

0.7508112258470164 0.9965258298439331 0.1032202221828076

0.8361077579035455 0.1672144837678718 0.1036690724581916

0.0029693561971985 0.2549146327452855 0.1038734009984488

0.0862244812752698 0.4227161163466295 0.1040869195178519

0.2601082510083999 0.2690016580304244 0.1033602323111062

0.5138802588490968 0.2691083946877222 0.1030708701813871

0.7569630133583745 0.2549242715927668 0.1036396556240682

0.8415032321046622 0.4226983073559098 0.1038656859247369

0.0065904634511461 0.5081543600330167 0.1042495556073506

0.0900977967893497 0.6751634183278349 0.1042726632500786

0.2512103713773710 0.5079280243425482 0.1036422565463140

0.3349787280345102 0.6754483555405838 0.1034275974883284

0.7617391684177988 0.5078800636335445 0.1032693560896156

0.8454981341427603 0.6753622433620527 0.1034022452577981

0.0104598394429953	0.7606149529243083	0.1039808504124156
0.0937182336240957	0.9284309880647643	0.1038391957317912
0.2551944558317433	0.7606682514630876	0.1039939094262595
0.3397402269826110	0.9284906565708291	0.1037131198695245
0.5828115171020855	0.9143362524035943	0.1029749935422254
0.8365789898813574	0.9143172154003982	0.1032399198787673
0.5745896578267594	0.6084811727199517	0.2581276067918990
0.3366009732882986	0.4319960048944135	0.1031119403014762
0.6004406565554307	0.4320355923519391	0.1024325687251733
0.4962655171381727	0.7514029792591466	0.1023085133531539
0.7600940466769908	0.7513010595441497	0.1029058052452630
0.6764882266337126	0.5791630998016419	0.2587839433717404
0.4730339138987218	0.6388408553536440	0.2592430855397449
0.1705038323917615	0.5271640651339167	0.3317621377174856
0.2593973460639313	0.7289145608313877	0.4376309534032069
0.2695542294010573	0.5756950867098565	0.5505579083925259
0.5483557243276513	0.5916790696591849	0.1021934980600681
0.2637719855449703	0.5543109721366399	0.3050950568694861
0.0993437805272141	0.5390566895942704	0.3015810296317673
0.2238320497454022	0.6565375769291999	0.3984463457906905
0.1827078194750350	0.7632681004473073	0.4423524521527891
0.2638937597683311	0.6328270764804771	0.5105439682073108
0.2716742939146544	0.6385267848982733	0.5885029164757596

$N_3C_1\_CO_2$ (0.5 charge)

1.0000000000000

9.8400001526000000   0.0000000000000000   0.0000000000000000
-4.9200000763000000   8.5216901053999994   0.0000000000000000
0.0000000000000000   0.0000000000000000   20.0000000000000000
C   N   O   Ni   H
28   3   5   1   6
Direct
0.0064928870974856 0.0097144800271822 0.1054147568042236
0.0904810114035402 0.1789200885281447 0.1054852487104659
0.2609577518468911 0.0184559794498744 0.1055840400799172
0.3454773705038991 0.1865927378683978 0.1052623052285411
0.5052644964791891 0.0091492182187689 0.1053533863734057
0.5906068352784455 0.1784958474491018 0.1052617825408853
0.7503214188784624 0.9988974412227553 0.1051662678929333
0.8352217074337464 0.1689044014725410 0.1054502705506113
0.0032990911307825 0.2574677354199632 0.1056812940371809
0.0852355548684861 0.4254238030289514 0.1059200104188677
0.2603615968883921 0.2699232067457951 0.1053008687148302
0.5130173851592081 0.2699682394596132 0.1050429294386069
0.7562672051346498 0.2567261985515203 0.1054243891156344
0.8401040692549885 0.4252973596876785 0.1056645289746711
0.0051341602033714 0.5107060135823803 0.1060963871222115
0.0892552116514422 0.6775138769054664 0.1061540260907865
0.2505212344445139 0.5119170009585360 0.1055791730420629
0.3342817004262226 0.6804221520178398 0.1054611844319086
0.7597309564314659 0.5096536457600638 0.1051944931836740
0.8453187534553802 0.6788849955217056 0.1053226255613323
0.0099305679973494 0.7629601196925737 0.1057941819352719
0.0932351813115219 0.9309783047590214 0.1056274290206701

0.2546610213775602	0.7639531362381060	0.1059472119004146
0.3399061880099773	0.9311823425450306	0.1056570604643500
0.5820802913856591	0.9164308783542099	0.1050715272166735
0.8372895174791487	0.9170450106912706	0.1052084907079157
0.5776977539863869	0.6116587540583281	0.2624050606700378
0.3394488710742938	0.4364987655959421	0.1051961644230390
0.5985965088959455	0.4339982459795210	0.1045937427565491
0.4944082426481792	0.7535098191690257	0.1047280430840293
0.7617083673088405	0.7555252629302432	0.1050190794546956
0.6643419949681223	0.5621943980716688	0.2616486424364620
0.4918410443849366	0.6623235941674683	0.2653526966577590
0.1709242316998142	0.5096276475370108	0.3260998569434090
0.2681210816237449	0.7233529868844654	0.4276036496911578
0.2553699846783380	0.5671025506389019	0.5431174822934346
0.5473942510878272	0.5923670691531503	0.1046195267146400
0.2571047409440148	0.5464087423021925	0.2941440516674653
0.0822544855149525	0.5008299437851991	0.3007451435775836
0.2283202789657575	0.6470084554511347	0.3898239172227052
0.1988487282765797	0.7672598530274526	0.4297381094571002
0.2593061516180953	0.6265751404999127	0.5021973817245555
0.2873715167923355	0.6429950855918674	0.5793043146674107

N_2C_2_CO_2 (0.5 charge)

1.00000000000000

9.8400001526000000	0.0000000000000000	0.0000000000000000
-4.9200000763000000	8.5216901053999994	0.0000000000000000
0.0000000000000000	0.0000000000000000	20.0000000000000000

C N O Ni H

29 2 5 1 6

Direct

0.0049749819450540	0.0089101769967286	0.1048597220667560
0.0891085150342263	0.1774031980409630	0.1049944506754270
0.2598773022049560	0.0180735534558778	0.1048855122638410
0.3439264305397100	0.1855586252215820	0.1046199873854050
0.5043896282174670	0.0086686623016519	0.1045881751665830
0.5897185234141770	0.1776377747821410	0.1044691262752480
0.7501705993137620	0.0007350929980419	0.1046606510622680
0.8342111685501190	0.1682396018758470	0.1049677253787710
0.0024327872594583	0.2566070974627480	0.1052040040226460
0.0845466079714896	0.4242425322559600	0.1053955517888810
0.2581945852961600	0.2682529611437300	0.1049593395971020
0.5123908036813920	0.2692694319284430	0.1041714939339610
0.7549358841503920	0.2557613159211340	0.1048167697355710
0.8394477974691340	0.4228005172049980	0.1050482017722560
0.0050150612207176	0.5098897094103950	0.1055119717306990
0.0890713067278455	0.6764051720108450	0.1054880645783310
0.2495869401326590	0.5100875548767549	0.1051602851997370
0.3342538342623840	0.6794949636063020	0.1048510644489650
0.7598309051549140	0.5068093451567820	0.1042889100293890
0.8444797276089280	0.6762166064578000	0.1044680606388230
0.0095372504271722	0.7620622706037070	0.1050533914349710
0.0916507518209286	0.9296926641372990	0.1050207127996150
0.2546510843928120	0.7635134792312210	0.1052564786478600
0.3391552789056930	0.9305444188874930	0.1048666624791820
0.5817069884310300	0.9170233252298270	0.1044259504736120
0.8358910479306020	0.9180232192265270	0.1046823745198110

0.5767182713477370 0.6081701471062080 0.2566081332729550

0.3375919211966120 0.4343404528155400 0.1050921546116470

0.7564360913863690 0.7519137397369420 0.1044383046723210

0.5991366364680319 0.4331551439062910 0.1035296472988850

0.4949517401035810 0.7531311711638370 0.1040361747067780

0.6587527835207750 0.5553002953942330 0.2613122645643320

0.4936321934807070 0.6629119918374400 0.2616512240068760

0.1671487789013000 0.5294236845672340 0.3171746787157230

0.2481281349048390 0.7056963144781320 0.4218753518860080

0.2579935442915800 0.5692442298609490 0.5354881153787050

0.5470421303808169 0.5931524976758310 0.1048684535112230

0.2875274916216950 0.5608160411451590 0.2915576811162590

0.1010012733283790 0.5158210292556050 0.2937876016599790

0.2146081839635060 0.6415680709137560 0.3785497910202340

0.1743467337285550 0.7363100558664830 0.4255314353371350

0.2526252281264550 0.6221594276398080 0.4940838050068520

0.2806088361859200 0.6419984827831871 0.5716236429855140

Bent_CO_2_Ni-N_4 (0.5 charge)

1.00000000000000

9.8400001526000000   0.0000000000000000   0.0000000000000000

-4.9200000763000000   8.5216901053999994   0.0000000000000000

0.0000000000000000   0.0000000000000000   20.0000000000000000

C   N   O   Ni   H

27   4   5   1   6

Direct

0.0051578215755007 0.0054743063410142 0.1027214123791483

0.0889611292528851	0.1744258145866011	0.1026383602925953
0.2591853086278296	0.0143460286966311	0.1027784548197710
0.3445339318313657	0.1849883824110068	0.1023888708178543
0.5045140493858999	0.0055343307162943	0.1021570070421212
0.5896615282873884	0.1744360504166145	0.1022910569678326
0.7495562599679404	0.9948415276564749	0.1021912592847259
0.8348834851758331	0.1654942406507767	0.1025914236132319
0.0017522683105929	0.2532321813922294	0.1025904435040743
0.0850463034812436	0.4210346309119695	0.1022132734607702
0.2587858791927273	0.2671689732401436	0.1022135086167751
0.5125218593025254	0.2671520118367053	0.1018478814052613
0.7557274765369401	0.2531729537958715	0.1023639826424849
0.8403353311538569	0.4209410865610295	0.1022806790978358
0.0054175215170276	0.5064805879226734	0.1023975641558313
0.0889045649065408	0.6734980111470733	0.1024470658076096
0.2500749786473380	0.5062601192196984	0.1013918502747587
0.3338655986340835	0.6738384619992041	0.1008929090091587
0.7604849638647195	0.5060689423430617	0.1014998165627425
0.8442231672692557	0.6735721453288338	0.1017915838143850
0.0091625507475635	0.7588412015951164	0.1024986440999177
0.0923548790083156	0.9266343498421701	0.1028151471250648
0.2539695040324946	0.7589745397733013	0.1020704686254132
0.3384706929002990	0.9267606629414581	0.1023847444422500
0.5815364749776174	0.9126676377434514	0.1013658596794843
0.8353310113862951	0.9127024351130912	0.1022716369166295
0.5637527398944562	0.5956276077423143	0.2317010356192140
0.3351626255769818	0.4300590265631633	0.1013490110081076
0.5992465524043908	0.4299732869030016	0.1005227491805455

0.4950466742668676 0.7500095865245476 0.0995027722232240
0.7590596323559973 0.7497092578962417 0.1016974656964815
0.6684720868028822 0.5725837122840137 0.2419716662275809
0.4583672669520544 0.6212567863656150 0.2347326510289156
0.1921939798951599 0.5451332910424803 0.2965775210034955
0.1819550931126811 0.6849207690124787 0.4079421478450403
0.2560733912161507 0.5757266639681332 0.5175480344603297
0.5473070538578859 0.5898462396819749 0.1012816907071443
0.2976008784924423 0.5616026384908226 0.2867642221564999
0.1772815490244041 0.6081526431421119 0.2629139003789095
0.1822561638837396 0.6282594405296306 0.3661287113477476
0.0749374502591769 0.6639201138246588 0.4135214983654842
0.2277777151035640 0.6153685945901658 0.4768990323364795
0.2444727117237607 0.6336182882717637 0.5543864204610796

TS01_Ni-N_4 (0.5 charge)
1.00000000000000
9.8400001526000000 0.0000000000000000 0.0000000000000000
-4.9200000763000000 8.5216901053999994 0.0000000000000000
0.0000000000000000 0.0000000000000000 20.0000000000000000
C N O Ni H
27 4 5 1 6
Direct
0.0051290541485151 0.0054632163201125 0.1033396654513052
0.0885634405183075 0.1744992372782977 0.1033088107162752
0.2592658576909780 0.0149796535186254 0.1032966392849257
0.3443151764330437 0.1850368793226067 0.1032933791150871
0.5039786542203820 0.0053636748138433 0.1031837898466221

0.5895812307630373	0.1743575582301590	0.1030513316747121
0.7492193658740793	0.9947117176672341	0.1033045962053091
0.8342185050722550	0.1647466713774061	0.1031272751167414
0.0012982727136367	0.2527962195708995	0.1028424611106575
0.0843610836630061	0.4206808893646876	0.1019610269299933
0.2579025246113780	0.2667406874873211	0.1033047275693343
0.5124595374528225	0.2665429875939092	0.1028155193772973
0.7551826785001972	0.2527800264601391	0.1027520074548835
0.8400223954725725	0.4206214215378076	0.1021654448754483
0.0050305994567418	0.5063916831101777	0.1019929718938802
0.0885072665758392	0.6734365960315168	0.1020994022631641
0.2493728644227284	0.5054071764154836	0.1013196231663396
0.3338758243114920	0.6744450430978159	0.1012866091291864
0.7594860074035696	0.5052014978715573	0.1015650944205679
0.8440588214028739	0.6743765441443175	0.1017797569772933
0.0091374974492701	0.7590807093482186	0.1024231373373382
0.0921852210147457	0.9269447031853104	0.1030997905086276
0.2535463701878240	0.7591422072800735	0.1020652268950890
0.3383820661119343	0.9269999039364933	0.1029096850118254
0.5810477217682364	0.9130979581764588	0.1031732225481707
0.8358050642472812	0.9131977192910331	0.1031661038611961
0.5539910965207198	0.5888274839983387	0.2103951347189416
0.3333815196550731	0.4294002626928915	0.1020812249364886
0.5995682920328473	0.4289940870196229	0.1011652694057984
0.4938956689838189	0.7504906249892905	0.1018578602349388
0.7604074764203919	0.7506622164074362	0.1017289338156292
0.6575964700377495	0.5832993497066800	0.2411025415637149
0.4310183512498441	0.5927955075290325	0.2357159831332818

0.1335085514821703 0.5410911513274651 0.2987274069733677

0.1797115355629379 0.6796157709239201 0.4110497121136865

0.2525641811689282 0.5762797443631995 0.5142507089479150

0.5469919690889973 0.5899228213466817 0.1122439098611402

0.4295443369575320 0.5863579202665414 0.2850364740004517

0.1911125081585843 0.6232139874357195 0.2660364330150222

0.1532442809789045 0.6002448819337041 0.3449117552782737

0.0799172247474892 0.6728774904342996 0.4211735919387589

0.2206483455554814 0.6193163277445877 0.4723938050925170

0.2455463371086432 0.6346126022860455 0.5519984548894181

Cis-COOH_Ni-N_4 (1.5 charge)

1.00000000000000

9.8400001526000000 0.0000000000000000 0.0000000000000000

-4.9200000763000000 8.5216901053999994 0.0000000000000000

0.0000000000000000 0.0000000000000000 20.0000000000000000

C N O Ni H

27 4 5 1 6

Direct

0.0020691780235861 0.0032077029875218 0.0988009599782199

0.0851461093431842 0.1718345742523300 0.0994774633210335

0.2561272504729730 0.0125888245963202 0.0986674256441368

0.3411618832611362 0.1826041648891200 0.0996176188684470

0.5007320082580394 0.0030342304875421 0.0993113091294899

0.5862849642552359 0.1716318863274783 0.0986674145886327

0.7458846540866649 0.9921090847026764 0.0995214579092432

0.8308706380220071 0.1620888757514159 0.0985832422140936

0.9979363239413083 0.2502810065527036 0.0984897577644383

0.0809267201846740	0.4181539685447809	0.0973297998492352
0.2541922966276186	0.2639922087107189	0.1003984585554931
0.5094800148696901	0.2637944089757817	0.0988327974606452
0.7519577833352236	0.2502384799763602	0.0977370640172126
0.8367949425609367	0.4180715101271986	0.0961119768166572
0.0017179467940667	0.5038475844235515	0.0957893486665318
0.0853103125450930	0.6710064040052857	0.0957644421875881
0.2459874225060980	0.5025855496489608	0.0985321800749670
0.3308038661317726	0.6721934620523404	0.0983995514139663
0.7560361576718320	0.5024820863364752	0.0959798505083756
0.8409618752343657	0.6722938767698615	0.0960733198014684
0.0061262688193448	0.7567334008935458	0.0962329909467785
0.0890872641281551	0.9245448674048125	0.0978929429945071
0.2502047967949258	0.7566518613469628	0.0971620075821031
0.3350704552677146	0.9244858273369888	0.0983966365292544
0.5774882258837700	0.9107517709682489	0.1001738764956301
0.8330776271323472	0.9109742946532098	0.0989249844501404
0.5646193870327076	0.5881051639406764	0.2097890774107310
0.3293764715986062	0.4262686369066626	0.1006533272164424
0.5964273579614253	0.4259167826911617	0.0972723010440261
0.4904839804362517	0.7485257416265280	0.1003928886023934
0.7579005460132547	0.7488227244633611	0.0974428368033783
0.6728944756295020	0.5848374274886102	0.2381136171733992
0.4473869084242306	0.5931681314750080	0.2431692123065305
0.1471729593815418	0.6155304825828690	0.2680107460323476
0.1382765736873895	0.6142303101325602	0.3971167182719404
0.2463771111460921	0.6024788288869324	0.5118611920385491
0.5443005090417898	0.5874013850451911	0.1134499167501923

0.4662015166480376 0.5938730971885892 0.2916773518255239

0.2371386085285866 0.6071876414679607 0.2535533928527553

0.1485558168631531 0.6142591030117629 0.3200963721623736

0.0380905833616823 0.6082377480034413 0.4054155975732612

0.1993960297298080 0.6074175827499857 0.4651312930427064

0.1787345733641954 0.6118830376165229 0.5446710381251701

Trans-COOH_Ni-N_4 (1.5 charge)

1.00000000000000

9.8400001526000000   0.0000000000000000   0.0000000000000000

-4.9200000763000000   8.5216901053999994   0.0000000000000000

0.0000000000000000   0.0000000000000000   20.0000000000000000

C   N   O   Ni   H

27   4   5   1   6

Direct

0.0024474405511276 0.0029155551911394 0.0951065806466260

0.0852623974534374 0.1711616979800201 0.0955128008080016

0.2563899528647458 0.0122054166205069 0.0946469867318570

0.3413933114171220 0.1821644623024297 0.0959293887399136

0.5010640571014517 0.0028348111297171 0.0953837876253492

0.5865237102793490 0.1710336073236780 0.0949570149876861

0.7461337974730402 0.9916661971077758 0.0960638693973414

0.8311430194338227 0.1616477106599928 0.0947848343411878

0.9981384654254902 0.2497954228151661 0.0943052619980509

0.0810557802914043 0.4176662588740393 0.0923365672621344

0.2539153993846069 0.2632140907811086 0.0964767113760409

0.5099660504602505 0.2631215663575184 0.0953366164429008

0.7522974157496736 0.2498184355616617 0.0938425654101980

0.8370454072618508	0.4176178388746070	0.0917272261215735
0.0019148730633982	0.5033310869913254	0.0907561519857620
0.0855506875111244	0.6706350463433574	0.0907236739053777
0.2461368351868377	0.5018244494482397	0.0936134415855770
0.3312198005062718	0.6720435775774799	0.0934937869835117
0.7559669305514178	0.5016814580084890	0.0921236797152251
0.8411795177906547	0.6721162421240067	0.0922191561866531
0.0063035601539885	0.7562826572579012	0.0917710862106473
0.0893485624121636	0.9240769718839809	0.0938753567350954
0.2503646335107982	0.7562729001457161	0.0921888847180625
0.3352910150823916	0.9241372187520677	0.0941006968091472
0.5775806117565522	0.9106934751651135	0.0964347927947430
0.8337961119101556	0.9107563888693978	0.0955689846716933
0.5639230112543282	0.5880510153186584	0.2094205734443164
0.3289034464085372	0.4251066631875017	0.0969067430505795
0.5967100280123961	0.4247889150418741	0.0943438464033211
0.4907226033859769	0.7488550033048693	0.0967754876059390
0.7588468520760578	0.7490317667263349	0.0946163726212847
0.6681915266221676	0.5788223868416493	0.2386533531056258
0.4545806944312671	0.6001459481882552	0.2478547832394586
0.4666353925805568	0.6260168102173737	0.3976963706073268
0.2012799197297198	0.5624640631120272	0.4486340311062320
0.2947586363925522	0.6366854294713158	0.5689698485657387
0.5445036374039661	0.5869489805928468	0.1140690864327272
0.4545924755496111	0.6125498550275992	0.3493285211656778
0.3777565380405100	0.6093504108194591	0.2201081077512161
0.3569746731356653	0.6013472602378112	0.4159702391326954
0.1745566360948824	0.6392257292053696	0.4323292424339270

0.2525843907330196 0.6062552230400167 0.5194046585955855
0.2025469145656318 0.5808575335205958 0.5971090855479276

Cis_COOH_Ni-N_2C_2 (charge 1.5)
1.00000000000000
9.8400001526000000   0.0000000000000000   0.0000000000000000
-4.9200000763000000   8.5216901053999994   0.0000000000000000
0.0000000000000000   0.0000000000000000   20.0000000000000000
C   N   O   Ni   H
29   2   5   1   6
Direct
0.0027485749044492 0.0026845978947644 0.1005310866511550
0.0870585548855951 0.1710642113961298 0.1007607351420849
0.2570198836689609 0.0107927993507016 0.0996123859329767
0.3416711002577138 0.1788796944819485 0.1016081648006967
0.5021334788257950 0.0023834395703988 0.0998565485190731
0.5875557585924638 0.1712012194234786 0.0995635919510718
0.7480377277180461 0.9947558303678946 0.1019641891580085
0.8326803824129142 0.1628423504198987 0.0998889018391809
0.0005324064147389 0.2506408059815006 0.0996031399963917
0.0828922603239859 0.4183403361346403 0.0976363235592266
0.2563999696261069 0.2617031291892775 0.1032835004004780
0.5098662458698350 0.2623248817444893 0.0999331125817597
0.7530382760868783 0.2496965616432182 0.0985310501005150
0.8373283986405837 0.4166503803584635 0.0969624546107265
0.0028718609801011 0.5034195323000579 0.0959741691163137
0.0868179094550535 0.6702313414460870 0.0958126664525580

0.2481416229395387	0.5046800432865173	0.1000053804791154
0.3321557510957752	0.6729507065891790	0.0985018085180277
0.7573389373884343	0.5006319222572040	0.0974429295110066
0.8414021215737822	0.6689656973564841	0.0989369168173167
0.0067697883525299	0.7552905362959494	0.0970284924154374
0.0891814995234043	0.9230215824613481	0.0991953062592093
0.2523614529665821	0.7569883464490447	0.0971657601371169
0.3366567230568441	0.9239246542085544	0.0985546188711123
0.5798406497387534	0.9112697388148639	0.1008845666245526
0.8333770421795124	0.9119842515468728	0.1029737319962825
0.5507951514539241	0.5801214308477198	0.2104060029053397
0.3354150873629798	0.4280987650445670	0.1063196712906311
0.7542580881062242	0.7456099368144551	0.1053648030713671
0.5960338040159836	0.4262749229351045	0.0975022693232612
0.4935327512687217	0.7471080703069299	0.0994851922657681
0.6273035688117421	0.5361917151363234	0.2430345215814922
0.4590807556554574	0.6310352685738784	0.2416308242065756
0.1412347063029765	0.6262018842339969	0.2668472816413532
0.1306849990402158	0.6269383177562753	0.3941641684580027
0.2485416005054591	0.5835853813727037	0.4974786924343121
0.5449744481808472	0.5866964833012221	0.1145471215386662
0.4712366373234703	0.6245062788034539	0.2902457595621860
0.2296983346724037	0.6159790957974858	0.2518324383625362
0.1431283454401043	0.6249909464639938	0.3195287617706719
0.0181870202366052	0.5690394383749701	0.4019046403149113
0.1968852418630330	0.6026627415576100	0.4531884373364822
0.2279015972814763	0.6379672437103429	0.5329935824950521

CO_Ni-N_4 (1 charge)

1.00000000000000

9.8400001526000000 0.0000000000000000 0.0000000000000000

-4.9200000763000000 8.5216901053999994 0.0000000000000000

0.0000000000000000 0.0000000000000000 20.0000000000000000

C N O Ni H

27 4 5 1 7

Direct

0.0033292158013244 0.0040751922942495 0.0957879601118834

0.0872317616009018 0.1736717295157806 0.0958151047950694

0.2578056234547652 0.0139602103190822 0.0960928107923721

0.3429667169494597 0.1842239037410354 0.0959415204859115

0.5024504632367424 0.0041258605663919 0.0958564654992288

0.5881699039127103 0.1737021763809811 0.0957081311512114

0.7475706978384729 0.9934044312424445 0.0958245616249515

0.8327109000347563 0.1636887029170561 0.0958427279470079

0.9997465327137605 0.2516905146547306 0.0958926966367144

0.0830838738128637 0.4199233709053526 0.0960416743479332

0.2570025847599716 0.2663349869473330 0.0958247395010206

0.5110059436716371 0.2662865793946950 0.0956813197125018

0.7536926316586694 0.2516764595259829 0.0957415436058788

0.8386186792738365 0.4199056449936969 0.0957927712650267

0.0036147299882358 0.5054596178638002 0.0960725416617650

0.0870431292973481 0.6723266620867177 0.0962231642745296

0.2477973173190634 0.5045604912146286 0.0960095264757678

0.3321137234570419 0.6732084716868727 0.0960980464968601

0.7585132290856132 0.5045203782656539 0.0955532598461381

0.8428059649255195 0.6730982657923477 0.0955849690703977

0.0075035114565636	0.7577836536981145	0.0960102337894322
0.0907927339250621	0.9259994945155470	0.0959903440692377
0.2520409345975430	0.7578531911629105	0.0962581802309138
0.3369425994341108	0.9260604057741321	0.0961105640957632
0.5794752977069519	0.9113823538384394	0.0958169613147281
0.8335538440247300	0.9113583692082150	0.0956750355206493
0.5616930717841970	0.5895619127496147	0.2249290427020907
0.3328454970327876	0.4290570785728777	0.0956581816270678
0.5979161142214365	0.4291150034176401	0.0953705531977905
0.4926322619913611	0.7486825461257035	0.0957123028012535
0.7576473528368168	0.7485449458060229	0.0953362184818297
0.6722665512203618	0.5929906583043039	0.2461278130153379
0.3952767926666873	0.6091766547911236	0.3836719120390140
0.2931722816930649	0.7695699543824890	0.3016702149658582
0.1851940798260069	0.8162813268740985	0.4087064914685731
0.2365585089932554	0.6501945263846076	0.4884617617164071
0.5453115371396994	0.5888618865787838	0.0976944703385431
0.3645710373767064	0.6622815235108378	0.3494696750889541
0.3435749262017253	0.6197242713113957	0.4246899136487041
0.2069310064956479	0.6943247248673763	0.2740399466591080
0.2401664076419202	0.7870960192990528	0.3449017835473423
0.0729412944616412	0.7764783666690280	0.4057525832123979
0.2111421332843557	0.7225661217279321	0.4558676408831683
0.3081081141946725	0.7208285201208987	0.5222132012837494

CO_N_2C_2 (0.5 charge)

1.00000000000000

9.8400001526000000 0.0000000000000000 0.0000000000000000
-4.9200000763000000 8.5216901053999994 0.0000000000000000
0.0000000000000000 0.0000000000000000 20.0000000000000000
C N O Ni H
29 2 5 1 7
Direct
0.0076983025362158 0.0052505425847635 0.0960050369181828
0.0920984250939399 0.1735263062254771 0.0960496713900221
0.2618449091302633 0.0135385888167224 0.0936746697992077
0.3465202726840158 0.1813916735786479 0.0971935297800810
0.5071284682295127 0.0050206987531881 0.0938906748653099
0.5927636826309043 0.1736956465967309 0.0932496312104865
0.7534555523210602 0.9974568936616822 0.0982305533808771
0.8380480069692829 0.1653231592219472 0.0941263438094419
0.0056934500722180 0.2532112157991222 0.0935702215900828
0.0882647720681051 0.4208152704461232 0.0901450758139218
0.2611898294366014 0.2638053981014683 0.0992039723628342
0.5152937295106435 0.2646783783000806 0.0949633696878408
0.7581196831462552 0.2522307871229150 0.0914492977976008
0.8423829679736283 0.4187600851769467 0.0885993261860162
0.0080395476769270 0.5057367047764808 0.0874075847295688
0.0919249785759972 0.6731504458223597 0.0874086608493265
0.2538560027619243 0.5073818788450591 0.0938012789326483
0.3381680612553479 0.6761069358937123 0.0924276797851740
0.7617207834033876 0.5027425588843737 0.0904223947350395
0.8459435759547663 0.6714953392863930 0.0924555412458069
0.0116307351294963 0.7581101525857276 0.0893823012949592
0.0941898877302267 0.9256851539532422 0.0931380334840484

0.2575427503305173 0.7600620072871334 0.0896270748479627

0.3418002518263675 0.9265953214992196 0.0918867340523390

0.5846153422084401 0.9140589008253622 0.0966046470335122

0.8386743916152145 0.9148845618977837 0.0996997246416149

0.5449971087277712 0.5752957768888110 0.2072619208000171

0.3396387613432470 0.4305039402556948 0.1024587038837363

0.7600882071714413 0.7480149441471778 0.1023555156722775

0.6014990197865984 0.4281586877835939 0.0937534607315002

0.4983672652740889 0.7505436617699369 0.0963650250907524

0.5378952729694598 0.5590905761810739 0.2645174776712464

0.3919946760004980 0.6095960577792567 0.4256165130326497

0.2886218260531709 0.7649651805845477 0.3450661217492860

0.1915106165164433 0.8213613877741548 0.4509806057169886

0.2411779451709705 0.6582151511913541 0.5320378658734659

0.5501272225418993 0.5890304761999432 0.1175822584559929

0.3446018538137859 0.6228579030273811 0.4673922921989556

0.3590522433008522 0.6622750999127706 0.3918662239276071

0.1992537451505543 0.6878149980488777 0.3195213295491218

0.2386808908967577 0.7879015491493504 0.3901480365571936

0.0796992135183467 0.7840579969130915 0.4488331207715648

0.2163584637381760 0.7312604971772828 0.4980990084325877

0.3133598247546819 0.7299610672730487 0.5652568336611650

TS13_Ni-N_4 (0.8 charge)

1.00000000000000

9.8400001526000000 0.0000000000000000 0.0000000000000000

-4.9200000763000000 8.5216901053999994 0.0000000000000000

0.0000000000000000 0.0000000000000000 20.0000000000000000

C N O Ni H

27 4 5 1 7

Direct

-0.0026375630520235 -0.0007172200273011 0.0983015582278175

0.0812421866277627 0.1688658771557177 0.0983672872597020

0.2520277209701897 0.0094077568395609 0.0983777267242740

0.3370971405020158 0.1795327086767402 0.0987723163216025

0.4964136208728775 -0.0007702391739396 0.0984608793677843

0.5821770318139062 0.1688464114688894 0.0983549274878307

0.7415888352125499 0.9885591394252410 0.0988740149563876

0.8266500492141537 0.1586611592580152 0.0984624369650119

0.9937525541580247 0.2468432879660456 0.0982078050395148

0.0770736577536697 0.4150866961857765 0.0978152478936981

0.2509644154388951 0.2615908502760186 0.0986342374887039

0.5053015017243623 0.2616206689536972 0.0984830300370060

0.7476508836659610 0.2467912222855868 0.0981954242171812

0.8325997745088226 0.4150113207372701 0.0976833821514625

-0.0023638090479717 0.5006613565875314 0.0975128353754778

0.0811076179868050 0.6675725083001997 0.0974255186954581

0.2418802496106672 0.4996263529696036 0.0980728958319614

0.3263308192029090 0.6685400394422033 0.0980750783228266

0.7523915852209264 0.4995864198805782 0.0976966307734559

0.8368451061665471 0.6685378698030464 0.0976782475270268

0.0016218271857935 0.7531355886266424 0.0976412819992322

0.0849186495097846 0.9213468563527913 0.0981185911036735

0.2461105008671189 0.7530975285393477 0.0976523728426142

0.3309744541867427 0.9212541182868013 0.0981331351849215

0.5733829196633645 0.9064794589681215 0.0988763506969442

0.8276779472729620 0.9064790111241636 0.0984887332460500

0.5747689924380647 0.5901852518153994 0.2340419944244355

0.3267808089741595 0.4242200368789347 0.0987086182990350

0.5921191952149815 0.4242291300159336 0.0982038492943930

0.4865875709534222 0.7438344089148482 0.0989937355177659

0.7518383405724959 0.7438361459902211 0.0981255147461225

0.7024084174619147 0.6360948959562543 0.2556040535257744

0.4525766188284442 0.6176507105103608 0.3012999999615705

0.2740902031530204 0.7214290487671937 0.2643693230651541

0.1929798694212332 0.8167057280413933 0.3725959371200500

0.2001353561511086 0.6566867416582773 0.4822438601138216

0.5393492959885302 0.5840551848031713 0.1023671486671128

0.5345986044226555 0.6898803770456415 0.3317573562689315

0.3497920780942513 0.6802588069595439 0.2787821511579535

0.1830755104145739 0.6313119441494520 0.2449164630420600

0.2198309443226129 0.7770381153741541 0.3305956388980955

0.0886269560845175 0.8016551494252663 0.3651097329181744

0.1981634699252279 0.7159565307829294 0.4420609136692066

0.2833629104820378 0.7347823932858916 0.5102372302722203

TS23 [1 charge] Ni-N_4

1.00000000000000

9.8400001526000000 0.0000000000000000 0.0000000000000000

-4.9200000763000000 8.5216901053999994 0.0000000000000000

0.0000000000000000 0.0000000000000000 20.0000000000000000

C N O Ni H

27 4 5 1 7

Direct

0.0120810148030324	0.0009107598171155	0.0947139948424755
0.0960069502254142	0.1704873486185230	0.0945502806568753
0.2667055292658235	0.0108870829089662	0.0944966666003223
0.3518673595783831	0.1811499191618281	0.0949357155521998
0.5112293360508701	0.0008542000916258	0.0946108927675113
0.5969190514715133	0.1704396899686683	0.0946057355056974
0.7563598148016941	0.9902075530028995	0.0951391045108294
0.8414592327221684	0.1604424815510721	0.0945676134259706
0.0084641931160963	0.2484776497377228	0.0942378934393626
0.0918596223642914	0.4167448375036275	0.0937078659890013
0.2657895794508161	0.2632358368580566	0.0947266787625983
0.5200280691450323	0.2632638318514641	0.0947633827112127
0.7624570920220954	0.2484610216195746	0.0943786723045137
0.8473682886971164	0.4167054472404364	0.0939083122957285
0.0123914221440974	0.5022889587575413	0.0934683516107044
0.0958484417263692	0.6692099269377125	0.0934293983421991
0.2566294627773154	0.5012542177044423	0.0940055651746490
0.3409889306133909	0.6699659799136781	0.0941452372253086
0.7672628174198548	0.5013104989821674	0.0942673746545078
0.8515906600583789	0.6700411951342981	0.0944516846802342
0.0163300600272166	0.7547223256313179	0.0939354055178961
0.0996604678938232	0.9229470305227857	0.0943684445909968
0.2608767956363741	0.7546190798310940	0.0937283097584501
0.3457078687344949	0.9228504532983113	0.0942651017708493
0.5882245228392046	0.9080759953629839	0.0949854604017301
0.8423409500896666	0.9080624409147561	0.0951179612791914
0.5591571623339460	0.5680661937625940	0.2413934374954096
0.3417335081524223	0.4258883352477303	0.0946170760519771

0.6067764059082612	0.4259359530514647	0.0947235474132652
0.5015299985001038	0.7452892649459515	0.0951200207232981
0.7662978613024325	0.7452725333685174	0.0953220531206080
0.6740916054042732	0.6159124522827664	0.2751970975709340
0.3942426494293136	0.5072110648148918	0.2956353246147229
0.3093177867974433	0.6812778400602255	0.3633599029720259
0.2598250134139417	0.8224429567426360	0.4690778496329958
0.3690737506466198	0.7320073572311293	0.5757364322036350
0.5540205533762464	0.5854931315640260	0.0978970917625215
0.3501409916229222	0.6157455735681371	0.3401334855903493
0.3114729633732284	0.4667265855341003	0.2619477418829990
0.2562940350711710	0.7045559173297550	0.3279510414455113
0.2842944200363960	0.7715610474287751	0.4314546753980705
0.3204671837179431	0.9347692727846135	0.4604123043566661
0.3268624408181747	0.7685926189650503	0.5389528650900463
0.3041380334981036	0.7202525714548264	0.6143714035905273

TS13 (1.0 charge) Ni-N_3C_1 system

1.00000000000000

9.8400001526000000	0.0000000000000000	0.0000000000000000
-4.9200000763000000	8.5216901053999994	0.0000000000000000
0.0000000000000000	0.0000000000000000	20.0000000000000000

C	N	O	Ni	H
28	3	5	1	7

Direct

0.0056104088228370	0.0050665281061676	0.1005449446913426
0.0897132435395745	0.1741201126680818	0.1009816637185093
0.2596887340522263	0.0133143501896071	0.1004251211616270

0.3445247807074939	0.1815638363556488	0.1024225297708979
0.5043872985250056	0.0046315598969453	0.1004084579453215
0.5897953485087806	0.1735575177743431	0.1004926201114563
0.7494106485505314	0.9942519884715758	0.1022549155748138
0.8345531833622585	0.1643110458568360	0.1006379005161063
0.0026757269830715	0.2529791929995346	0.1000189724732340
0.0846317874563879	0.4207154493019894	0.0983305665896283
0.2593120827306422	0.2647776182513267	0.1027433114180803
0.5119578338166257	0.2645395758684170	0.1017062356848727
0.7555360000033782	0.2519551687604155	0.0996659872089097
0.8394810126824072	0.4205330179303050	0.0980283137642031
0.0043586903935093	0.5057974239157876	0.0970753046098639
0.0885262830876453	0.6729334763603065	0.0968126685280765
0.2503646907243527	0.5075804009824123	0.0997990598016248
0.3338480759093216	0.6760521496240658	0.0993511976895081
0.7584898415751028	0.5046923610465390	0.0988525048542465
0.8440247796662391	0.6739882207870884	0.0988373867357404
0.0090819936719175	0.7581274160536845	0.0977234354851453
0.0922173286426315	0.9259921829660889	0.0995779149456066
0.2536412274076150	0.7593853134534531	0.0977678068518692
0.3387961914536430	0.9262044074780338	0.0993524512620744
0.5811715240776354	0.9123332518230558	0.1017428375806948
0.8366808355918287	0.9125775185613443	0.1017044006497951
0.5696541239868083	0.5898176844114349	0.2180159309622209
0.3378057854746322	0.4311799525738596	0.1040174550845736
0.5977299008036293	0.4280485799844200	0.1015440241886486
0.4929669428777622	0.7498270448744403	0.1020575940471991
0.7616140373408780	0.7513457505416806	0.1020100644771947

0.6482432270610469 0.5599066868036101 0.2523548024098846

0.3768840137058348 0.5534789702247749 0.2794072996999797

0.2287375867441497 0.6944911493083314 0.2673960838995691

0.2097740338248400 0.8195454762675281 0.3784551571349570

0.2247666473254092 0.6616460182234102 0.4862137244818792

0.5463114237059736 0.5868933252384128 0.1143638654524286

0.4375989039115896 0.5908117097148383 0.3205419328964514

0.2959284596098931 0.6317282149774318 0.2720854877916610

0.1276505079524857 0.6200922231519221 0.2482158274098534

0.2116071477869906 0.7663130428147842 0.3344997701448892

0.1126332414282099 0.8228454501864076 0.3778573246768133

0.2183294707190901 0.7206517875826932 0.4466986774875874

0.2714354011391129 0.7396888757511457 0.5214671731144193

TS13_Ni-N_2C_2 (1 charge)

1.00000000000000

9.8400001526000000   0.0000000000000000   0.0000000000000000

-4.9200000763000000   8.5216901053999994   0.0000000000000000

0.0000000000000000   0.0000000000000000   20.0000000000000000

C   N   O   Ni   H

29   2   5   1   7

Direct

0.0075681664647540 0.0028502585984881 0.1005848439501345

0.0918318583495176 0.1711656527040165 0.1016471691632067

0.2617863635652728 0.0112215457989449 0.0992941365658379

0.3461138110654670 0.1787431277157367 0.1028683819595660

0.5069418418380400 0.0026660299583631 0.1000432111345821

0.5923355537743981 0.1710606451850431 0.0990276713443600

0.7532001362505133	0.9951471205988254	0.1038174663816785
0.8375230753985113	0.1627127869922261	0.1001921568275803
0.0056004207366549	0.2509581873307427	0.0999034126265237
0.0880031868980955	0.4183491599862204	0.0971851263055581
0.2611548231757457	0.2617326696377837	0.1053139029886419
0.5149460119754092	0.2621979987757097	0.1002674294957124
0.7579144150474092	0.2498099561487986	0.0973068956672649
0.8420673562275927	0.4162311819741434	0.0936845136334325
0.0080335887078499	0.5034695970451745	0.0933573451311085
0.0916821467715873	0.6704867109456083	0.0929993715638578
0.2537551396236820	0.5049439405580853	0.1013491310895318
0.3378099786148614	0.6735641665046657	0.0995279755169705
0.7616015224142177	0.5003048435411055	0.0943167901306692
0.8456978897625875	0.6690363159842250	0.0960632568163959
0.0116535012408437	0.7557869509295813	0.0938959167161201
0.0937795275388058	0.9230767286238633	0.0979646604595016
0.2574712115793351	0.7575674380136195	0.0958908060995838
0.3414316729323011	0.9240396783851607	0.0979605226214914
0.5842289742100573	0.9114596359007356	0.1027556079520439
0.8382806922125472	0.9121937087646993	0.1041409273293005
0.5829434703536719	0.5691967687973049	0.2111507909898738
0.3396128554340095	0.4279796680849276	0.1097214463514442
0.7600679583285702	0.7457309164981892	0.1060192427690177
0.6009078806845997	0.4254518374863506	0.0980473547143966
0.4983465495758244	0.7482009754532282	0.1032024966587857
0.6297257147269620	0.5391113902349501	0.2586806893974133
0.4143785835980938	0.6473081939199404	0.3270331928470052
0.2168589456898269	0.7281416556284360	0.2875320845572713

0.1893801476450041	0.8108467544913708	0.4002864934237427
0.2216629093422320	0.6538734596703926	0.4944451572260909
0.5511041952005615	0.5866095889056852	0.1230751380377116
0.3938937841112126	0.6301526239083663	0.3746935932859209
0.3323207939359162	0.6758125561419664	0.3102189323666361
0.1189852052763076	0.6362127406388776	0.2744794410464987
0.1960034830882877	0.7660265490542881	0.3427305642921487
0.0886830896193694	0.8060195807534549	0.4041463103829500
0.2080152671339758	0.7186041680987784	0.4564499793946707
0.2874718094375057	0.7309864156843571	0.5275357692929127
